# Low-dose docetaxel reprograms CAR T cells for enhanced solid tumor immunity

**DOI:** 10.1186/s13046-026-03781-9

**Published:** 2026-07-15

**Authors:** Songshan Zhu, Jun Yin, Xin Fu, Weiqiang Yang, Yiwei Zeng, Rui Chen, Na Huang, Ling Zhang, Cong Yu, Qianying Huang, Ping Ouyang, Kaisong Huang, Hangjiu Su, Dan Jiang, Guangxian Xu

**Affiliations:** 1 Guangdong Provincial Key Laboratory of Medical Immunology and Molecular Diagnostics, The First Dongguan Affiliated Hospital, School of Medical Technology, Guangdong Medical University, Dongguan, People’s Republic of China; 2 Dongguan Key Laboratory of Molecular Immunology and Cell Therapy, Guangdong Medical University, Dongguan, People’s Republic of China; 3 Institute of Transplant Medicine, The Second Affiliated Hospital of Guangxi Medical University, Guangxi Clinical Research Center for Organ Transplantation. Guangxi Key Laboratory of Organ Donation and Transplantation, Nanning, People’s Republic of China; 4 Department of Laboratory Medicine, The People’s Hospital of Guangxi Zhuang Autonomous Region, Nanning, People’s Republic of China

**Keywords:** CAR T cell, Solid tumor, Docetaxel, Exosomes, Tumor microenvironment, Immunotherapy

## Abstract

**Background:**

Chimeric antigen receptor (CAR) T cell therapy in solid tumors is hampered by dense stromal barriers, rapid functional exhaustion, and limited persistence. Here, we report that low-dose docetaxel acts as an immunomodulatory primer that reprograms CAR T cells to address these barriers. Retrospective analysis of breast cancer patients identifies host lymphocyte reserve as a key determinant of docetaxel treatment benefit, implying an immunological component beyond direct tumor cell killing.

**Methods:**

Sub-cytotoxic docetaxel concentrations were evaluated in primary human T cells and CAR T cells using phenotypic, transcriptional, and functional assays. Secretory profiles and extracellular vesicle cargo were characterized, with efficacy assessed in 3D spheroids and xenograft models.

**Results:**

Docetaxel priming induces a "Metabolic-Cycle Uncoupling" state, constraining proliferation while enhancing cytotoxic effector potency and memory traits. This reprogramming is associated with upregulation of Rab27-dependent exosome biogenesis, accompanied by shedding of exhaustion markers (PD-1, CD57) and enrichment of cytotoxic ligands (FasL, TRAIL), CAR, and homing receptors (CCR5/CCR7). Primed CAR T cells exhibit improved antitumor efficacy with associated stromal remodeling and host T cell activation; secreted exosomes contribute to bystander tumor cell killing.

**Conclusions:**

These findings describe a non-genetic pharmacologic priming strategy with potential scalability and compatibility with existing manufacturing processes. This approach demonstrates enhanced CAR T cell performance against solid tumor barriers in preclinical models, highlighting the potential of chemotherapy-immunotherapy synergies.

**Graphical Abstract:**

Low-dose docetaxel acts as an immunomodulatory primer that reprograms CAR T cells without impairing cellular viability. Sub-cytotoxic concentrations induce cell-cycle arrest with enhanced effector potency and memory traits, redirecting resources toward increased secretory activity. This promotes Rab27-dependent exosome biogenesis, with shedding of exhaustion markers (PD-1, CD57) and enrichment of cytotoxic ligands (FasL, TRAIL), CAR, and homing receptors (CCR5/CCR7). Primed CAR T cells exhibit improved antitumor efficacy with associated stromal remodeling and host T cell activation; secreted exosomes contribute to bystander tumor cell killing in spheroid and xenograft models.

**Figure:**
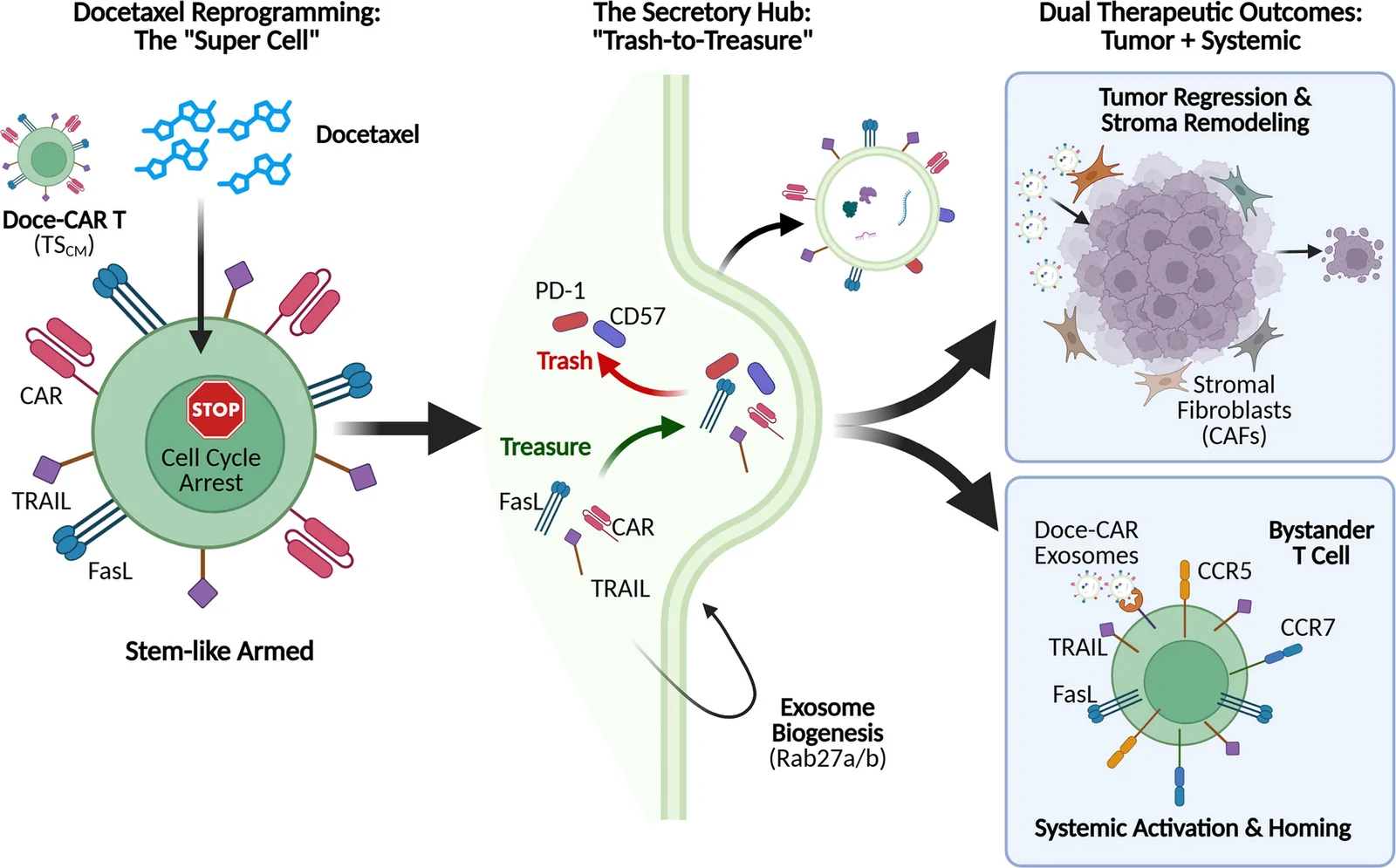

**Supplementary Information:**

The online version contains supplementary material available at https://doi.org/10.1186/s13046-026-03781-9.

## Background

Chimeric antigen receptor (CAR) T cell therapy has achieved transformative clinical success in hematologic malignancies; however, its efficacy in solid tumors remains limited. This failure is largely attributed to the hostile tumor microenvironment (TME), which imposes multiple layers of restriction on T cell function. Dense desmoplastic stroma physically excludes effector cells, metabolic stress accelerates functional exhaustion, and the infused products frequently lack durable stem-like persistence [1–3]. Together, these barriers restrict infiltration, limit longevity, and constrain antitumor activity.

To overcome these challenges, most current strategies focus on increasingly complex genetic engineering approaches, including checkpoint disruption and the enforced expression of cytokines or costimulatory molecules [4, 5]. While mechanistically appealing, such interventions introduce substantial manufacturing complexity, regulatory burden, and potential genotoxic risk. Importantly, many of these approaches act downstream of fundamental physiological constraints that govern T cell fate, raising the question of whether alternative, non-genetic strategies might achieve similar functional gains with greater translational feasibility.

One underexplored possibility lies in the immunomodulatory effects of conventional chemotherapeutic agents. Taxanes such as docetaxel are classically viewed as cytotoxic drugs that inhibit microtubule dynamics and suppress rapidly proliferating cells, including lymphocytes [6]. However, emerging clinical observations suggest that this view may be incomplete. In a retrospective analysis of breast cancer patients treated with docetaxel, we observed that therapeutic benefit was not solely associated with tumor characteristics, but was strongly stratified by the host immune status. Specifically, patients with a favorable neutrophil-to-lymphocyte ratio (NLR) exhibited significantly prolonged survival, indicating that treatment efficacy may depend on the availability of a functional lymphocyte pool. These findings suggest a substrate-dependent component to docetaxel efficacy, in which immune competence influences therapeutic outcome.

This observation prompted a key hypothesis: if host immune status constrains the efficacy of taxane-based therapy, could the drug itself be leveraged to directly modulate T cell fate? Rather than acting exclusively as a cytostatic agent, we asked whether low-dose docetaxel might exert a selective, non-lethal stress that reshapes T cell differentiation and function.

Here, we report that exposure to low-dose docetaxel induces a distinct functional state in CAR T cells characterized by "Metabolic-Cycle Uncoupling"—constraining proliferative progression while maintaining cytotoxic function and stem-like memory traits. This reprogramming is mechanistically linked to a shift toward Rab27-dependent secretory activity. Specifically, we show that this pathway serves as a dual-function quality control mechanism: actively shedding exhaustion markers (PD-1, CD57) to preserve cellular fitness, while concurrently deploying extracellular vesicles enriched in cytotoxic ligands and CAR-associated components.

Consequently, this non-genetic pharmacological strategy produces CAR T cells with enhanced functional properties, including evidence of stromal remodeling and bystander antitumor effects in preclinical models. This approach provides a scalable and integrable framework to enhance CAR T cell efficacy in solid tumors without additional genetic manipulation.

## Methods

### Patient cohort and clinical data analysis

The study protocol was approved by the Institutional Review Board (IRB) of The People’s Hospital of Guangxi Zhuang Autonomous Region (Approval No. KY-ZC-2023–003) and conducted in strict accordance with the Declaration of Helsinki. We retrospectively analyzed a cohort of 98 patients with histologically confirmed breast cancer who received docetaxel-based chemotherapy regimens between January 2010 and December 2024. Patients with active autoimmune diseases, concurrent infections, or incomplete medical records were excluded from the analysis.

Clinicopathological data, including age, tumor stage (TNM classification), and specific treatment regimens, were extracted from electronic medical records (Supplemental Tables 1–3). Peripheral blood indices were derived from complete blood counts (CBC) obtained within 14 days prior to the initiation of chemotherapy. The NLR was calculated as the absolute neutrophil count divided by the absolute lymphocyte count; the Lymphocyte-to-Monocyte Ratio (LMR) was calculated similarly.

Overall survival (OS) was defined as the time interval from the date of the first docetaxel dose to the date of death from any cause or the last follow-up. Survival curves were estimated using the Kaplan–Meier method, and differences between groups were assessed using the log-rank test. Univariate and multivariate Cox proportional hazards regression models were utilized to evaluate independent prognostic factors, with results expressed as hazard ratios (HRs) and 95% confidence intervals (CIs). All statistical analyses were performed using R software version 4.5.

### Cell lines and culture conditions

The tumor cell lines HCT116, MKN45, MCF7, NCI-H460, and HEK293T, as well as Jurkat T cells and cancer-associated fibroblasts (CAFs), were obtained from the Chinese Academy of Sciences. All cell lines were authenticated and routinely confirmed negative for mycoplasma contamination. HCT116, MCF7, CAFs, and HEK293T cells were cultured in high-glucose DMEM (Gibco, Cat# 10566016) supplemented with 10% Fetal Bovine Serum (FBS; Gibco, Cat# 10091148) and 1% Penicillin/Streptomycin (NCM, Cat# C100C5). MKN45, NCI-H460, and Jurkat T cells were maintained in RPMI 1640 Medium (Gibco, Cat# 11875093) supplemented with 10% FBS and 1% Penicillin/Streptomycin. All cells were maintained at 37 °C in a humidified incubator containing 5% CO₂. Adherent cells were passaged twice weekly using 0.25% Trypsin–EDTA (Gibco, Cat# 25200056).

### Human samples

Peripheral blood samples were obtained from healthy volunteers between June 2024 and August 2025. Written informed consent was obtained from all donors prior to collection. All procedures involving human specimens were approved by the Ethics Committee of The First Dongguan Affiliated Hospital of Guangdong Medical University and conducted in accordance with the Declaration of Helsinki.

### Primary human T cell isolation and culture

Peripheral blood mononuclear cells (PBMCs) were isolated from buffy coats of healthy donors via density gradient centrifugation using a lymphocyte separation medium (TBD, Cat# LTS1077) according to the manufacturer’s protocol. Primary T cells were purified from PBMCs by negative selection using the EasySep™ Human T Cell Isolation Kit (STEMCELL Technologies, Cat# 17951). Following isolation, T cells were activated with ImmunoCult™ Human CD3/CD28/CD2 T Cell Activator (STEMCELL Technologies, Cat# 10990) for 24–72 h. Activated T cells were subsequently transduced with lentiviral vectors as described below. T cells were maintained in X-VIVO 15 medium (Lonza, Cat# 04-418Q) supplemented with 1% Penicillin–Streptomycin-Glutamine (Gibco, Cat# 15140122), 50 µM β-mercaptoethanol (Gibco, Cat# 21985023), and 10 ng/mL recombinant human IL-2 (PeproTech, Cat# 200–02-50UG). Cells were cultured at 37 °C in a humidified atmosphere containing 5% CO₂.

### RNA sequencing and bioinformatics analysis

Total RNA was isolated from approximately 5 × 10^6^ CAR T or docetaxel-primed CAR T (Doce-CAR T) cells using TRIzol Reagent (Invitrogen, Cat# 16096040) according to the manufacturer’s instructions. RNA integrity and concentration were assessed using an Agilent 2100 Bioanalyzer (Agilent Technologies). For library preparation, 1 µg of total RNA was used as input. Poly(A) mRNA enrichment was performed using Oligo(dT) magnetic beads, followed by fragmentation using divalent cations at high temperature. First-strand cDNA was synthesized using random primers and reverse transcriptase, followed by second-strand cDNA synthesis where dTTP was replaced by dUTP to preserve strand specificity. Following end repair, A-tailing, and adaptor ligation, the libraries were amplified by PCR and purified. The libraries were sequenced on an Illumina NovaSeq 6000 platform to generate 150 bp paired-end reads (2 × 150 bp). Raw sequencing reads (FASTQ) were processed using Cutadapt (v.1.9.1) to remove adaptor sequences and low-quality bases (Phred quality score < 20). Clean reads were aligned to the human reference genome (GRCh38) using the splice-aware aligner HISAT2 (v.2.2.1). Gene-level read counts were quantified using HTSeq (v.0.6.1) based on the Ensembl gene annotation. Differential expression analysis was performed using DESeq2 (v.1.34.0). Genes with an adjusted p-value (FDR) < 0.05 and an absolute log_2_ (fold change) ≥ 1 were considered significantly differentially expressed. For functional enrichment, Gene Set Enrichment Analysis (GSEA) was performed using gseapy (v.1.1.9) against the MSigDB Hallmark gene sets, with a significance threshold of FDR < 0.25. Gene Ontology (GO) enrichment analysis was conducted using GOSeq (v.1.34.1), and KEGG pathway enrichment was assessed using hypergeometric testing. Visualization of data was performed using R software (v.4.5.1).

### Tumor spheroid formation

Tumor spheroids were generated by seeding 5 × 10^3^ cells per well into 96-well Ultra-Low Attachment (ULA) U-bottom plates (S-bio, Cat# MS-9384UZ). Cells were suspended in 180 µL of RPMI 1640 medium supplemented with 10% FBS and 1% Penicillin–Streptomycin. To minimize evaporation, plates were sealed with Breathe-Easy semipermeable membranes (Diversified Biotech, Cat# BEM-1). Spheroids were cultured at 37 °C in a humidified atmosphere containing 5% CO_2_.

### Spheroid viability and metabolic activity assay

To quantify tumor viability, intracellular ATP levels were measured using the CellTiter-Glo® 3D Cell Viability Assay (Promega, Cat# G9683). Following a 72-h co-culture with T cells, spheroids were carefully washed twice with PBS to remove residual suspension effector cells and minimize background luminescence. Individual spheroids were subsequently transferred to white opaque 96-well microplates (Thermo Fisher Scientific, Cat# 236108) in 100 µL of PBS. An equal volume of CellTiter-Glo 3D reagent was added to each well. Plates were shaken orbitally for 5 min in the dark to induce cell lysis and stabilize the luminescent signal, followed by a 25-min incubation at room temperature (20 ℃−25 ℃). Luminescence was quantified using a BioTek Synergy H1 microplate reader. Relative viability was calculated by normalizing relative luminescence units (RLU) to untreated control spheroids.

### Absolute quantitative metabolic flux assay

To quantify extracellular nutrient turnover, single-cell glucose consumption and lactate production rates were measured using the Glucose-Glo™ Assay (Promega, Cat# J6021) and Lactate-Glo™ Assay (Promega, Cat# J5021), respectively. CAR T cells were initially seeded at a density of 1 × 10⁶ cells/mL in 24-well plates and treated with either PBS (vehicle control) or 10 nM docetaxel for 72 h. Following this initial incubation, cells were harvested and washed twice with PBS to thoroughly remove residual docetaxel and accumulated background metabolites. Viable cells were precisely determined, and an equal number of 2 × 10⁶ viable cells from each group was re-suspended in fresh X-VIVO 15 medium and re-seeded into 48-well plates for a strict 6-h metabolic assay window. Supernatants were subsequently collected and cleared of cellular debris via sequential centrifugation at 300 × g for 5 min followed by 2,000 × g for 5 min. Cleared supernatants were serially diluted (1000-fold for glucose analysis, 50-fold for lactate analysis) in specified diluents and transferred to white opaque 96-well microplates in 50 µL volumes. An equal volume of the respective Glucose Detection Reagent or Lactate Detection Reagent was added to each well. Plates were incubated at room temperature (20 ℃–25 ℃) for 60 min in the dark to stabilize the bioluminescent signals. Luminescence was quantified using a microplate reader. Absolute metabolite concentrations were interpolated via high-fidelity standard curves (R^2^ > 0.999). To completely decouple metabolic rates from the confounding effects of differential cell proliferation during the assay window, functional metabolic fluxes were mathematically normalized to the exact 6-h duration and the integral of the viable cell count (calculated as the mean of the initial seeding number and the final viable cell count). Final metabolic influx and efflux values were expressed as nanomoles per million cells per hour (nmol/10⁶ cells/hr).

### Generation of lentiviral vectors and transduction of T lymphocytes

The Mesothelin (MSLN)-targeted CAR was designed as a classical second-generation construct comprising an anti-MSLN single-chain variable fragment (scFv, derived from Anetumab), a CD8 hinge and transmembrane domain, a 4-1BB costimulatory domain, and a CD3ζ signaling domain. Lentiviral particles encoding this specific CAR construct were commercially synthesized and packaged by GENEWIZ (Suzhou, China).To generate MSLN-CAR T cells, primary human T cells were activated and subsequently transduced using an optimized spinoculation protocol adapted from previously described methods [7]. Briefly, activated T cells were resuspended in basal X-VIVO 15 medium and seeded in 96-well plates at a density of 4 × 10^5^ cells/well. Lentiviral particles were added at a multiplicity of infection (MOI) of 8. Spinoculation was performed at 1,000 × g for 10 min at 32 °C. Following centrifugation, the plates were incubated at 37 °C with 5% CO_2_. At 12 h post-transduction, fresh medium was supplemented, and cells were subsequently expanded under standard conditions. CAR expression efficiency on the T cell surface was validated by flow cytometry three days post-transduction. To generate Doce-CAR T cells, the expanded CAR T cells were cultured in the presence of 10 nM docetaxel for 72 h in vitro. Following this ex vivo priming phase, the cells were thoroughly washed with PBS to remove any residual drug prior to their utilization in subsequent functional assays or in vivo adoptive transfer.

### Spheroid volume quantification and 3D rendering

Spheroid volumes were calculated using the open-source software AnaSP (v2.0) based on [brightfield/fluorescent] images. Three-dimensional (3D) structural reconstruction and surface rendering were performed using ReViSP (v2.3) to visualize morphological integrity [8, 9].

### Quantification of cytokine secretion

To assess functional cytokine release, 1.5 × 10^5^ CAR T cells were co-cultured with individual target tumor spheroids in 96-well ULA plates. Co-cultures were maintained in 180 µL of medium without exogenous cytokines for 72 h. Following incubation, supernatants were harvested and centrifuged at 300 × g for 5 min to remove cellular debris. The concentrations of IL-2, IL-4, IL-6, IL-10, TNF-α, and IFN-γ were quantified using the Six-Cytokine Detection Kit (WELLGROW, Cat# 281401HN) based on the multiplex bead-based flow immunofluorescence method. Data were acquired using an Agilent NovoCyte D3000 flow cytometer and analyzed with FlowJo v10.8.1 software.

### Exosome-induced T-cell migration and invasion assay

To evaluate the potential of exosomes to facilitate T cell infiltration through an extracellular matrix, a Transwell invasion assay was performed using 24-well plates (Falcon, Cat# 353504) equipped with 8.0-µm pore size cell culture inserts (Falcon, Cat# 353097). The upper surface of the insert membrane was coated with diluted Matrigel matrix (Corning, Cat# 354234) and incubated at 37 °C for 1 h to allow solidification, followed by a wash with PBS.

T cells were fluorescently labeled with CellTracker™ Red CMTPX (Thermo Fisher Scientific, Cat# C34565) according to the manufacturer’s instructions. Labeled T cells were resuspended in serum-free medium at a density of 5 × 10^5^ cells/mL. For the assay, 100 µL of the T cell suspension was added to the upper chamber. To assess exosome-mediated effects, 25 µg of Doce-CAR T-derived exosomes were simultaneously added to the upper chamber. The lower chamber was filled with 650 µL of RPMI 1640 complete medium supplemented with 20% FBS and recombinant human IL-2 to serve as a chemoattractant. After a 24-h incubation at 37 °C, T cell transmigration was evaluated. Cells that had migrated to the lower chamber were imaged using fluorescence microscopy and quantified using a hemocytometer.

### Isolation and purification of exosomes

To generate exosome-producing cells, CAR T cells were labeled with an APC-conjugated anti-G4S antibody (Clone B02H1, Hycells, Cat# APS240612) for 1 h at 4 °C in the dark. CAR-positive cells were sorted using a Fusion Cell Sorter (Aurora CS, Cytek). Sorted cells were cultured at a density of 2 × 10⁶ cells/mL for 48 h in X-VIVO 15 medium supplemented with 1% Penicillin–Streptomycin-Glutamine, 50 µM β-mercaptoethanol, 10 ng/mL recombinant human IL-2.

Exosomes were isolated from T cell culture supernatants via differential ultracentrifugation. Conditioned media were harvested after 48–72 h and centrifuged at 300 × g for 5 min to pellet cells, 2,000 × g for 10 min to remove dead cells, and 16,500 × g for 45 min to discard cellular debris. The clarified supernatants were then ultracentrifuged at 100,000 × g for 2 h at 4 °C using an Optima XPN-100 Ultracentrifuge (Beckman Coulter, Optima XPN-100). The resulting exosome pellets were washed by resuspension in PBS and collected by a second ultracentrifugation step at 100,000 × g for 2 h. Purified exosomes were resuspended in PBS and stored at − 80 °C until further analysis [10–12]. Particle size and zeta potential were analyzed using size and zeta potential analyzers (NanoBrook 90Plus, Brookhaven). For phenotypic analysis by flow cytometry, exosomes were coupled to 4 µm aldehyde/sulfate latex beads via overnight incubation. Exosome-bound beads were blocked with glycine and stained with fluorophore-conjugated antibodies against exosomal markers (CD63, CD9, CD81), T cell markers (CD3, CD4, CD8), and specific surface antigens (FasL, TRAIL, CAR, CD95, CCR7, CD45RA, CD27, PD-1, CD28, CD57, HLA-DR, CCR5). Samples were acquired using a NovoCyte Opteon Spectral Flow Cytometer (Agilent) and analyzed with FlowJo software (v10.8.1).

### Exosome internalization and T cell proliferation assays

To visualize exosome uptake, Doce-CAR T-derived exosomes were labeled with the lipophilic membrane dye PKH26 (BestBio, Cat# BB-441125) following the manufacturer’s instructions. To remove excess unbound dye, labeled exosomes were washed twice with PBS using 100 kDa Amicon Ultra-0.5 Centrifugal Filter Units (Merck Millipore, Cat# UFC5100BK) by centrifugation at 10,000 × g for 10 min.

For internalization assays, recipient cells (1 × 10^5^ HCT116 cells or 2 × 10^5^ T cells) were labeled with 5 µM CellTracker™ Green CMFDA (Thermo Fisher Scientific, Cat# C2925) for 40 min at 37 °C. Cells were gently washed twice with PBS to remove excess dye and subsequently co-cultured with PKH26-labeled Doce-CAR T exosomes. After a 12-h incubation, cells were fixed with 4% paraformaldehyde and nuclei were counterstained with DAPI. Confocal images were acquired using a Stellaris 5 confocal microscope (Leica Microsystems). To quantify uptake efficiency, recipient cells were analyzed by flow cytometry after 12 h of exposure to the labeled exosomes.

For the T cell proliferation assay, T cells were labeled with 5 µM CellTracker™ Green CMFDA prior to exosome treatment. Labeled T cells were incubated with Doce-CAR T exosomes for 3 days. T cell proliferation was determined by flow cytometry based on the dilution of CMFDA fluorescence intensity.

### Assessment of exosome accumulation in tumors

To evaluate in vivo tumor-targeting capacity, Doce-CAR T exosomes were labeled with the near-infrared fluorescent lipophilic dye DiR (Invitrogen, Cat# D12731) according to the manufacturer’s instructions. DiR-labeled exosomes were injected intravenously (via the tail vein) into HCT116 tumor-bearing NOD scid gamma (NSG) mice. The accumulation of labeled exosomes in tumor tissues was monitored at 3, 12, and 24 h post-injection using the AniView100 Multi-Model In Vivo Animal Imaging System (Biolight Biotechnology).

### Flow cytometric analysis

Surface staining of cells was performed using the following antibodies: anti-CD3 (UCHT1), anti-CD8 (SK1), anti-CD4 (SK3), anti-CD45RA (HI100), anti-CD197 (2-L1-A), anti-CD28 (CD28.2), anti-CD27 (L128), anti-CD95 (DX2), anti-CD57 (NK-1), anti-PD-1 (NAT105), anti-CD195 (2D7), anti-G4S (B02H1), anti-MSLN (EPR19025-42), GLUT1 (202,915). For the intracellular staining, cells were fixed, permeabilized, and stained with anti-Perforin (B-D48), anti-TNF-α (MAb11), anti-GzmB (QA16A02), anti- IFN-γ (4S.B3), HK1(EPR10134(B)) as described before [13].

### Establishment of xenograft models and In Vivo treatment regimens

Animal experiments were conducted in the Laboratory Animal Unit of Guangdong Medical University under specific pathogen-free (SPF) conditions. All procedures were approved by the Institutional Animal Care and Use Committee (IACUC).

To establish a low effector-to-target (E:T) ratio stress model, 1 × 10^6^ MSLN⁺ Luciferase⁺ (Luc⁺) HCT116 cells were injected subcutaneously into the flank of NSG mice. Tumor engraftment was monitored seven days post-inoculation by intraperitoneal injection of D-luciferin (150 mg/kg) followed by bioluminescence imaging (BLI) using the IVIS Lumina Series III system (PerkinElmer). Based on total flux (photons/s), mice were randomized into four groups to ensure equivalent baseline tumor burden. Each mouse subsequently received 1 × 10^7^ T cells, CAR T cells, or Doce-CAR T cells intravenously via the tail vein. Tumor progression was quantified as average radiance (p/s/cm^2^/sr).

To evaluate the generalizability of docetaxel priming, an independent xenograft model was established by injecting 2 × 10⁶ HGC27 cells subcutaneously into NSG mice. Treatment commenced 14 days post-inoculation, at which point mice were randomized and administered the respective T-cell therapies as described above.

To comprehensively evaluate both long-term therapeutic efficacy and the dynamic remodeling of the TME, a split-cohort design was employed (*n* = 12 mice per treatment group). One cohort (*n* = 6) was designated for long-term survival and longitudinal tumor growth monitoring, strictly adhering to established humane endpoint criteria. Because the HCT116 solid tumor model is highly prone to severe ulceration and central necrosis at advanced stages, the remaining cohort (dissection cohort, *n* = 6) was preemptively euthanized at a pre-designated intermediate endpoint (Day 30 post-treatment). This ensured the harvest of high-quality, viable tumor tissues devoid of severe necrotic artifacts for accurate flow cytometric and immunohistochemical (IHC) profiling. At these respective endpoints, peripheral blood, spleens, and major organs were harvested for downstream analysis. Furthermore, to eliminate potential analytical bias arising from macroscopic tumor size discrepancies between treatment groups at Day 30, we employed normalized quantitative strategies: flow cytometric results were reported as relative cell frequencies within the viable cell populations derived from enzymatically digested tumor tissues, and IHC quantification was performed by calculating the average positivity rate (%) across 10 randomly selected fields of view (FOVs) strictly localized within viable tumor parenchyma. Local cytokine levels within the TME were assessed in tumor tissue homogenates as described above.

### In Vivo evaluation of exosome efficacy

To evaluate the therapeutic efficacy of exosomes, HCT116 tumor-bearing mice were treated with Doce-CAR T-derived exosomes. Following tumor cell inoculation, mice received intravenous injections of exosomes (100 µg/mouse) or an equal volume of PBS every three days. Mice were monitored daily for clinical signs of morbidity (e.g., ruffled fur, weight loss, reduced activity). Tumor volume was measured using calipers, and overall survival was recorded.

### In Vivo assessment of exosome-mediated T cell modulation

To assess the impact of exosomes on T cell migration, 1 × 10^7^ T cells were injected intravenously into each tumor-bearing mouse. Subsequently, mice received intravenous injections of exosomes (100 µg/mouse) or PBS every four days. Tumor progression was quantified as average radiance (p/s/cm^2^/sr). At the experimental endpoint, spleens and major organs were harvested for histological and immunophenotypic analysis. Local cytokine levels within the TME were assessed in tumor tissue homogenates as described above.

### Histological and immunohistochemical analysis

Tissue samples were harvested in accordance with the study design. At the experimental endpoints, the spleen, lung, liver, brain, kidney, and heart were dissected, dehydrated, and embedded in paraffin. Sections were subsequently processed for Hematoxylin and Eosin (H&E) staining and IHC. Detailed information regarding the antibodies used for IHC is provided in the Key Resources Table.

### Isolation of single-cell suspensions from tumors and spleens

Tumor tissues and spleens were harvested from the respective treatment groups for flow cytometric analysis. Tumor specimens were minced into fragments of approximately 1–2 mm^3^ and enzymatically digested in 1 mg/mL Collagenase IV (Biosharp, Cat# BS165-100 mg) and 0.1 mg/mL DNase I (MCE, Cat# HY-108882-100 mg) for 30 min at 37 °C. The resulting digest was filtered through a 100-µm cell strainer (Biosharp, Cat# BS-100-CS-N2) to obtain a single-cell suspension. Samples were centrifuged at 300 × g for 5 min. Following the removal of the supernatant and lysis of red blood cells, the cell pellets were washed with cold PBS.

### Statistical analysis

Data points compromised by technical artifacts (e.g., presence of debris or sample loss during transfer) were manually excluded prior to analysis. Subsequently, outliers were identified and removed using the ROUT method (Q = 1%). Consequently, the final number of data points plotted in graphs may differ from the total number of technical replicates. All statistical analyses were performed using GraphPad Prism v10. The normality of data distribution was assessed using the Shapiro–Wilk test. For comparisons between two groups, an unpaired Student’s t-test was used. For comparisons among three or more groups, data were analyzed using a one-way ANOVA with Tukey’ s multiple comparison test (for normally distributed data) or the Kruskal–Wallis test with Dunn’ s multiple comparison test (for non-normally distributed data). All data are presented as mean ± SEM unless otherwise stated.

#### Key resources table

**Table:** 

Reagent for IHC	Source	Cat. no
CD3	Servicebio	GB12014
Ki67	Servicebio	GB111499
Collagen VI	Abcam	ab182744
Fibronectin α-SMA	Abcam Abcam	ab2413 ab7817
CD146	Abcam	ab75769
HRP conjugated Goat Anti-Mouse IgG HRP conjugated Goat Anti-Rabbit IgG HRP conjugated Goat Anti-Rat IgG	Servicebio Servicebio Servicebio	GB23301 GB23303 GB23302

## Results

### Therapeutic benefit of docetaxel relies on host lymphocyte reserve

**Fig. 1:**
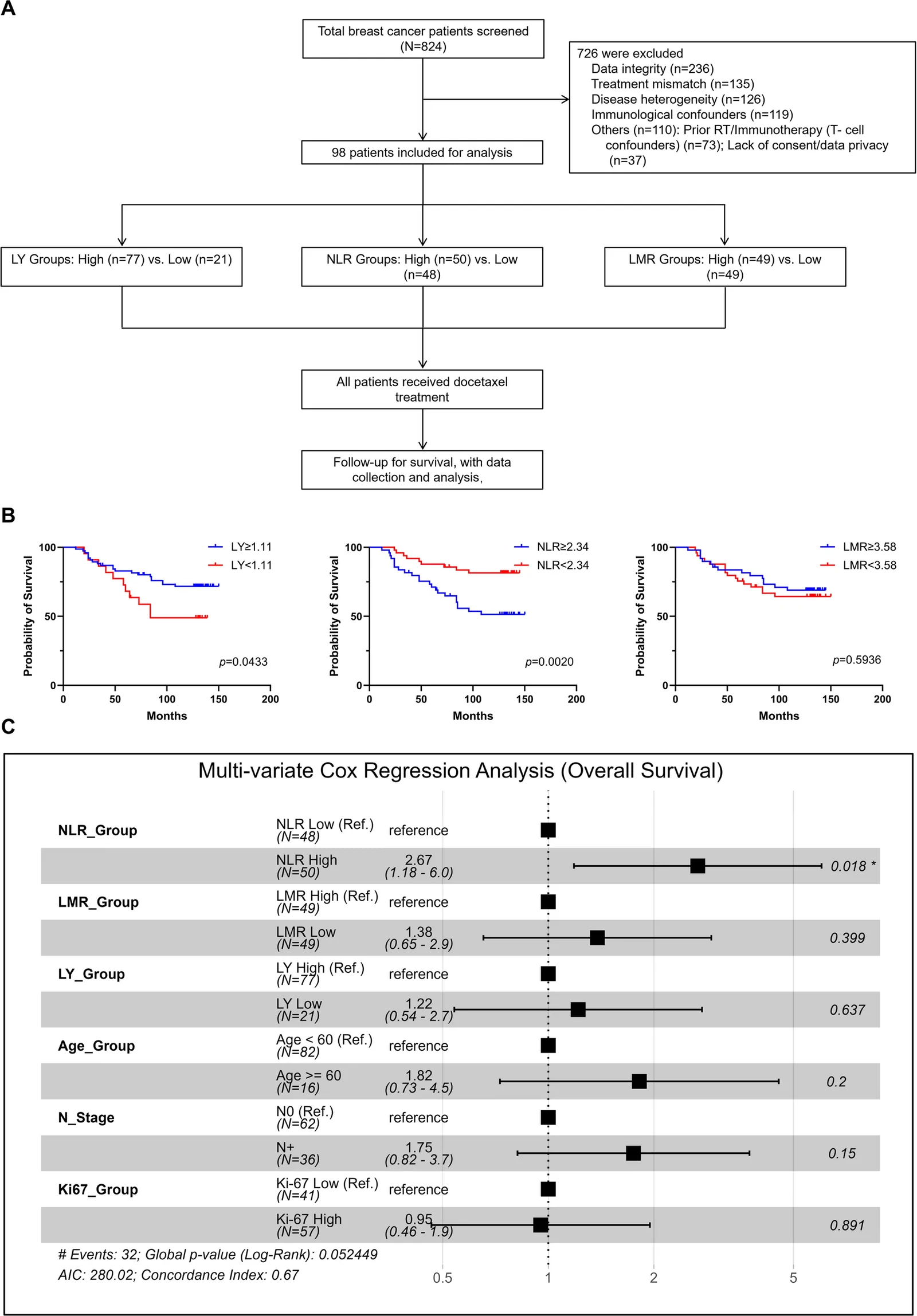
Systemic lymphocyte reserve is associated with therapeutic efficacy in Docetaxel-treated breast cancer. **A** Flow diagram of the retrospective patient cohort (*n* = 98), detailing enrollment, exclusion criteria, and stratification based on baseline immune signatures: absolute lymphocyte count (LY), neutrophil-to-lymphocyte ratio (NLR), and lymphocyte-to-monocyte ratio (LMR). **B** Kaplan–Meier estimates of overall survival (OS) stratified by baseline LY, NLR, and LMR status. *p*-values were determined by the Log-rank test. **C** Forest plot showing hazard ratios (HR) and 95% confidence intervals (CI) from the multivariate Cox regression analysis, assessing independent prognostic factors while adjusting for key clinical covariates (Age, N stage, and Ki-67)

To examine the relationship between systemic immune composition and clinical outcome, we analyzed pretreatment peripheral blood indices from 98 breast cancer patients receiving docetaxel-based therapy (Fig. 1A). Baseline clinicopathological variables were comparable across groups (Supplemental Tables 1–3).

Survival analysis revealed that the NLR was a strong prognostic factor. Patients with low NLR (≤ 2.34) showed prolonged overall survival compared with those with high NLR (116 vs. 93 months; *p* = 0.036; Supplemental Table 2). Kaplan–Meier curves confirmed this separation (*p* = 0.0020; Fig. 1B), with lower mortality in the low NLR group (19% vs. 46%; *p* = 0.004; Supplemental Table 2).

Absolute lymphocyte count was also associated with survival (*p* = 0.043), whereas LMR showed no significant association (*p* = 0.5936; Fig. 1B). Multivariate Cox regression identified high NLR as an independent predictor of poor survival (hazard ratio = 2.67; *p* = 0.018; Fig. 1C). These results suggest an association between systemic lymphocyte representation and docetaxel treatment outcomes, consistent with reported immunomodulatory effects of taxanes [14–16]. While this association does not establish causality and may reflect multiple biological mechanisms, it is consistent with the hypothesis that lymphocyte biology contributes to taxane efficacy, prompting evaluation of the direct effects of low-dose docetaxel on T cell function.

### Low-dose docetaxel enhances effector functions while preserving proliferative competence in primary T cells

**Fig. 2:**
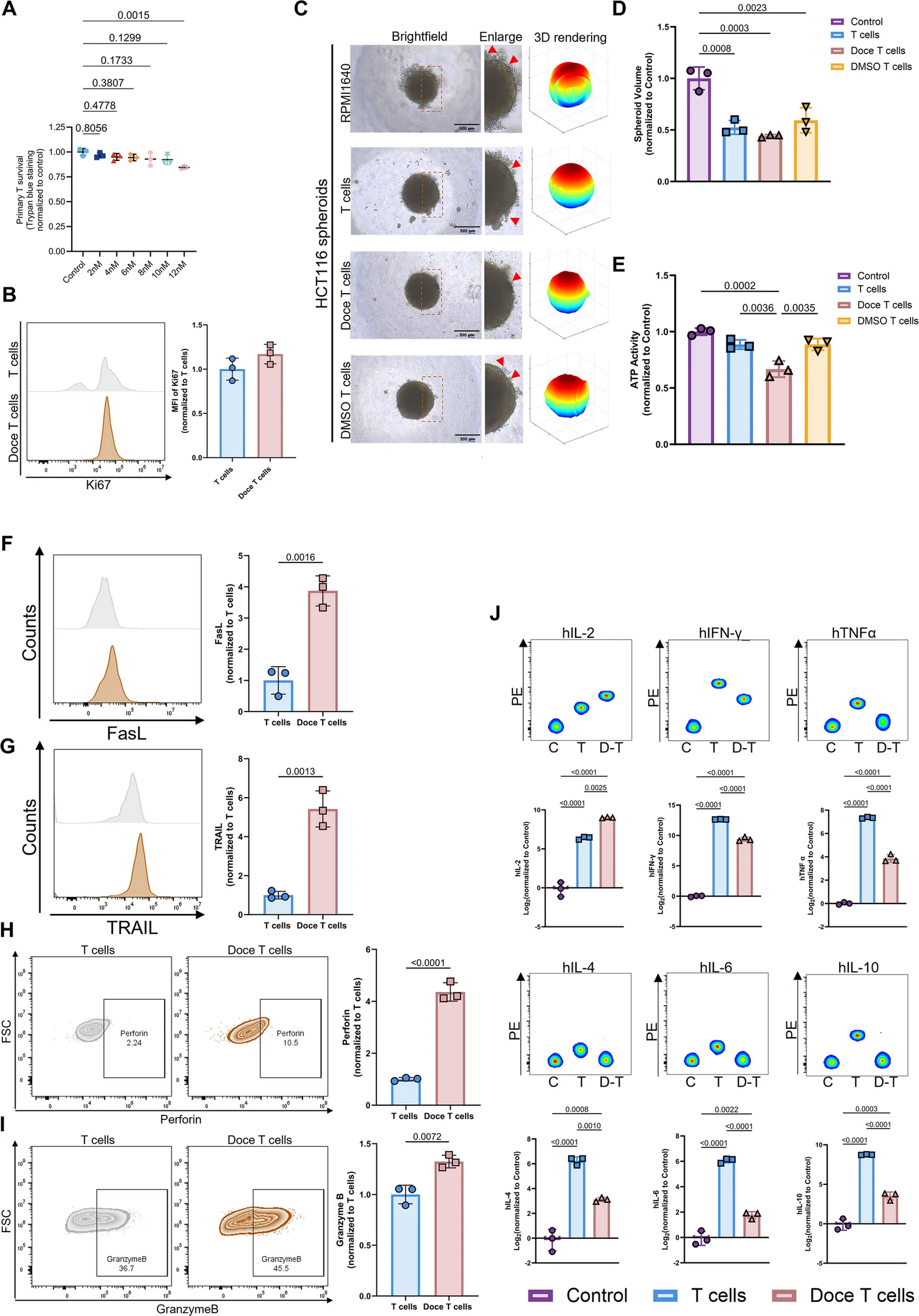
Characterization of cell viability, proliferative competence, cytotoxicity, and secretory profiles of primary human T cells under low-dose Docetaxel priming conditions. **A**Viability analysis of primary T cells treated with a gradient of Docetaxel (2–12 nM), assessed by Trypan blue exclusion. **B** Representative flow cytometric histograms (left) and quantitative analysis of the relative mean fluorescence intensity (MFI) (right) of the proliferation marker Ki67 in control primary T cells versus Docetaxel-primed T cells. **C** Representative brightfield microscopy and 3D volumetric rendering of HCT116 tumor spheroids following co-culture with control, unprimed (T cells), or Docetaxel-primed (Doce T) cells. Red arrows indicate structural disintegration. Scale bar = 500 μm. **D**, **E** Quantification of relative spheroid volume (**C**) and tumor metabolic viability assessed by ATP luminescence (**D**). **F**-**I** Flow cytometric plots and quantification of surface death ligands FasL (**F**) and TRAIL (**G**), and intracellular lytic markers Perforin (**H**) and Granzyme B (**I**). **J** Multiplex bead-based quantification of cytokine secretion levels in culture supernatants. For panels **A**–**E** and **J**, *n* = 3 biological repeats; data are presented as mean ± SEM. Statistical significance between groups was determined by one-way ANOVA followed by Tukey’s multiple comparison test or unpaired Student’s t-test (**B**, **F**-**I**)

To test whether docetaxel modulates T cell function at non-cytotoxic concentrations, we performed dose-titration experiments in primary human T cells. Cell viability was maintained at concentrations up to 10 nM, with no significant difference from untreated controls (*p* > 0.12), whereas exposure to 12 nM reduced viability (*p* = 0.0015; Fig. 2A). Consistent with these viability profiles, flow cytometric analysis revealed that Ki67 expression was robustly maintained between control and docetaxel-primed T cells, indicating preserved proliferative competence under low-dose priming conditions (Fig. 2B). A concentration of 10 nM was therefore used for subsequent in vitro experiments.

T cell activity was evaluated in a three-dimensional HCT116 tumor spheroid model that recapitulates aspects of solid tumor architecture (Fig. 2C). Compared with untreated T cells, docetaxel-treated T (Doce-T) cells induced greater disruption of spheroid structure, reflected by reduced spheroid volume (*p* = 0.0003; Fig. 2D) and decreased tumor metabolic activity by ATP content (*p* = 0.0002; Fig. 2E).

Phenotypic analysis indicated increased expression of effector molecules after docetaxel exposure. Flow cytometry showed higher frequencies of FasL⁺ T cells (approximately six-fold; *p* = 0.0016) and TRAIL⁺ T cells (approximately 20-fold; *p* = 0.0013; Fig. 2F, G). Intracellular staining revealed elevated Perforin (approximately four-fold; *p* < 0.0001) and Granzyme B (*p* = 0.0072; Fig. 2H, I).

Cytokine profiling revealed increased IL-2 secretion in Doce-T cells (*p* = 0.0025; Fig. 2J) and reduced IL-10 (*p* < 0.0001). Secretion of interferon-γ (INF-γ) and tumor necrosis factor-α (TNF-α) was lower compared with untreated controls.

These results indicate that low-dose docetaxel enhances cytotoxic effector pathways and alters cytokine output in primary T cells, with increased tumor disruption in a 3D model. We next examined whether these effects extend to engineered T cells and investigated the underlying mechanisms in CAR T cells.

To determine whether the observed immunomodulatory effects extend beyond docetaxel, we performed parallel experiments using paclitaxel. Low-dose paclitaxel preserved T-cell proliferative competence, as evidenced by unchanged Ki67 expression in viable T cells (Fig. S1A, B). Furthermore, paclitaxel-primed T cells exhibited enhanced disruption of 3D HCT116 tumor spheroids, closely mirroring the augmented cytotoxicity observed with docetaxel-primed cells (Fig. S1C). These findings indicate that the non-cytotoxic potentiation of T-cell effector function is a conserved pharmacological property of taxanes, consistent with prior reports [17], and further substantiate the link between host lymphocyte reserve and taxane-based therapeutic outcomes.

### Docetaxel alters differentiation and transcriptional programs in CAR T cells

To assess whether docetaxel exposure is associated with changes in CAR T cell state, we first used reporter Jurkat cells for initial validation [18]. MSLN-targeted CAR Jurkat cells were generated (Fig. S1D). Dose titration identified 850 pM docetaxel as the highest concentration maintaining viability (Fig. S1E). At this dose, docetaxel-treated CAR (Doce-CAR) Jurkat cells showed increased antigen-dependent cytotoxicity against MSLN⁺ HCT116 targets in 2D cultures (Fig. S1F, G) and 3D spheroids (Fig. S1H), with no activity against antigen-negative HEK293T spheroids (Fig. S1I).

Effects were then evaluated in primary human CAR T cells. Lentiviral transduction achieved efficiency exceeding 98% (Fig. S2A). Exposure to 10 nM docetaxel maintained viability, consistent with observations in unmodified T cells (Fig. S2B) [17]. This concentration was used for phenotypic and transcriptional analyses.

**Fig. 3:**
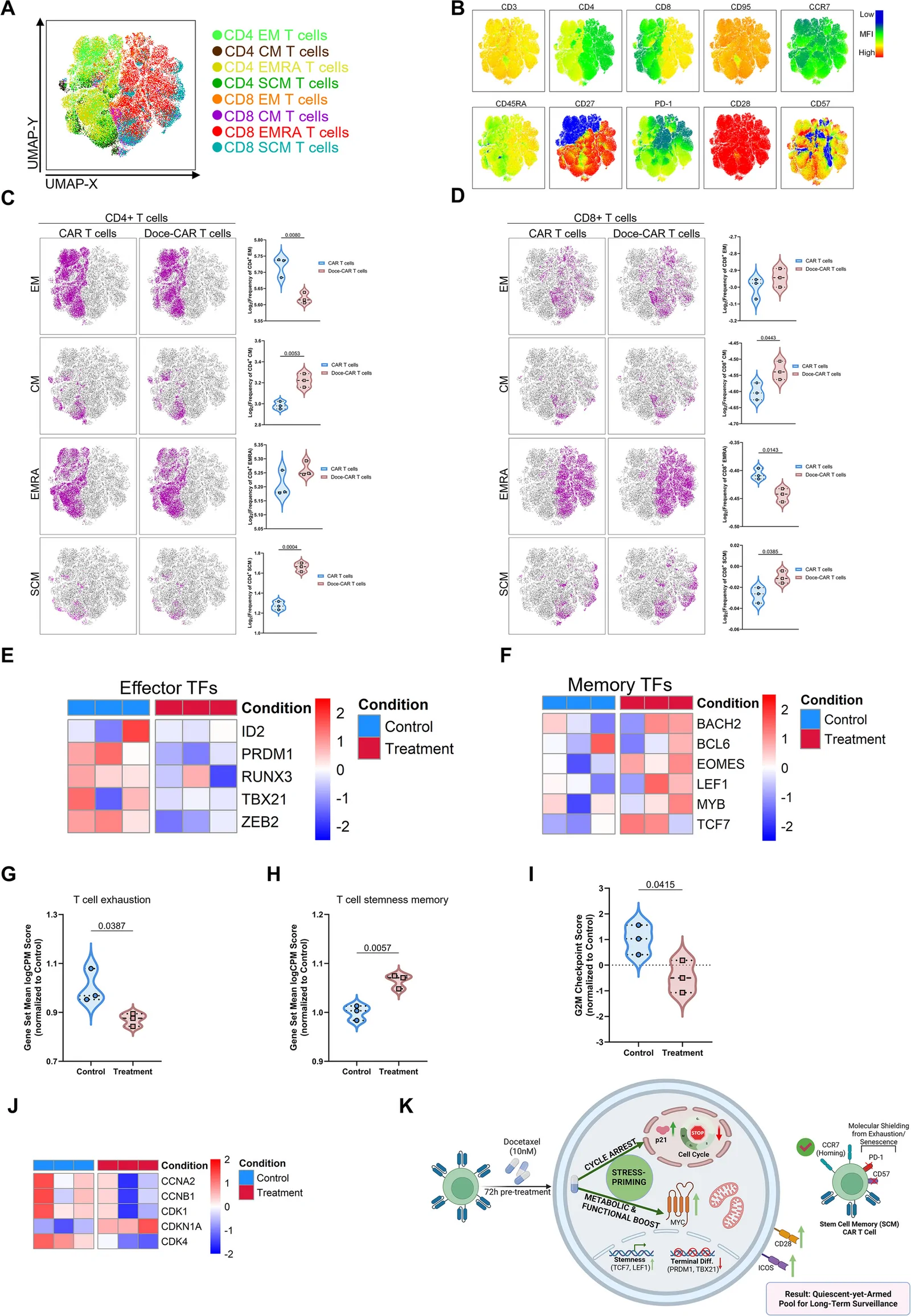
Phenotypic and transcriptomic characterization of CAR T cells following low-dose docetaxel exposure. **A** Uniform Manifold Approximation and Projection (UMAP) visualization of MSLN-CAR T cells, identifying eight distinct clusters based on high-dimensional spectral flow cytometry. **B** Feature plots projecting the expression intensity of canonical lineage (CD3, CD4, CD8) and differentiation markers (CCR7, CD45RA, CD27, PD-1, CD28, CD57) onto the UMAP space. **C**, **D** Violin plots quantifying the frequency of specific differentiation subsets within the CD4 + (**C**) and CD8 + (**D**) lineages: Effector Memory (EM), Central Memory (CM), Effector Memory Re-expressing CD45RA (EMRA), and Stem Cell Memory (SCM). **E**, **F** Heatmaps derived from RNA-seq data displaying the differential expression (LogCPM) of key transcription factors associated with effector differentiation (**E**) and stemness/memory maintenance (**F**). **G**, **H** Gene set expression scores derived from bulk RNA-seq data, calculated as the normalized mean logCPM of genes within T cell exhaustion (**G**) and stemness-memory (**H**) signatures in docetaxel-primed versus control cells. **I** Violin plot quantifying the GSVA enrichment score for "G2M Checkpoint". **J** Heatmap displaying the relative expression (LogCPM) of core cell cycle regulators (CCNA2, CCNB1, CDK1, CDKN1A, CDK4). **K** Schematic model of the proposed Stress-Priming mechanism. Prior to all phenotypic and functional analyses, MSLN-CAR T cells were primed with 10 nM docetaxel for 72 h. The diagram illustrates the proposed uncoupling effect whereby docetaxel-induced p21 upregulation arrests cell-cycle progression while preserving metabolic and functional competence. This rewiring limits terminal differentiation, reduces markers of exhaustion (PD-1) and senescence (CD57), and promotes the generation of a durable SCM-enriched phenotype. (For **C**, **D**, **G**, **H**, and **I**, data are presented as the mean ± SEM of *n* = 3 biological replicates. Statistical significance was determined by unpaired Student’s t-test

High-dimensional profiling revealed changes in differentiation-associated phenotypes following treatment. Uniform Manifold Approximation and Projection (UMAP) analysis identified eight clusters (Fig. 3A, B). Doce-CAR T cells showed increased proportions of stem cell memory (SCM) and central memory (CM) subsets, alongside alterations in effector memory (EM) and reduced terminally differentiated effector memory (EMRA) populations (Fig. 3C, D) [19, 20]. Bulk RNA sequencing indicated elevated expression of transcription factors associated with memory states (TCF7, LEF1) and reduced expression of genes linked to terminal differentiation (PRDM1, TBX21; Fig. 3E, F). GSEA showed reduced exhaustion signatures (*p* = 0.0387) and enrichment of stem-like features (*p* = 0.0057; Fig. 3G, H). Doce-CAR T cells also displayed downregulation of G2M checkpoint signatures (Fig. 3I), accompanied by upregulation of CDKN1A (p21) and suppression of proliferative drivers, including cyclins and CDKs (Fig. 3J).

Additional analysis revealed increased expression of genes involved in metabolic activity and costimulatory signaling, including MYC, CD28, and ICOS (Fig. S2C), as well as homing receptors CCR7 and SELL (Fig. S2D–I). To exclude transient activation delay, we evaluated CD25 and CD69 at the end of ex vivo expansion (Days 10–12), after docetaxel was introduced post-CAR transduction. Doce-CAR T cells maintained activation marker expression comparable to control CAR T cells (Fig. S2J–M), supporting functional remodeling rather than delayed activation. In addition, Doce-CAR T cells showed reduced expression of inhibitory and senescence markers, including PDCD1, CTLA4, and B3GAT1 (CD57; Fig. S3A). This was confirmed at the protein level by decreased PD-1 and CD57 surface expression in CD4⁺ and CD8⁺ subsets (Fig. S3B–I).

These data suggest that docetaxel treatment is associated with a redistribution toward memory-associated phenotypes, reduced exhaustion and senescence markers, and coordinated transcriptional changes in CAR T cells (Fig. 3K). We next investigated the impact on secretory activity and extracellular vesicle function.

### Doce-CAR T cells display enhanced and partly antigen-independent cytotoxicity with restrained proliferation

**Fig. 4:**
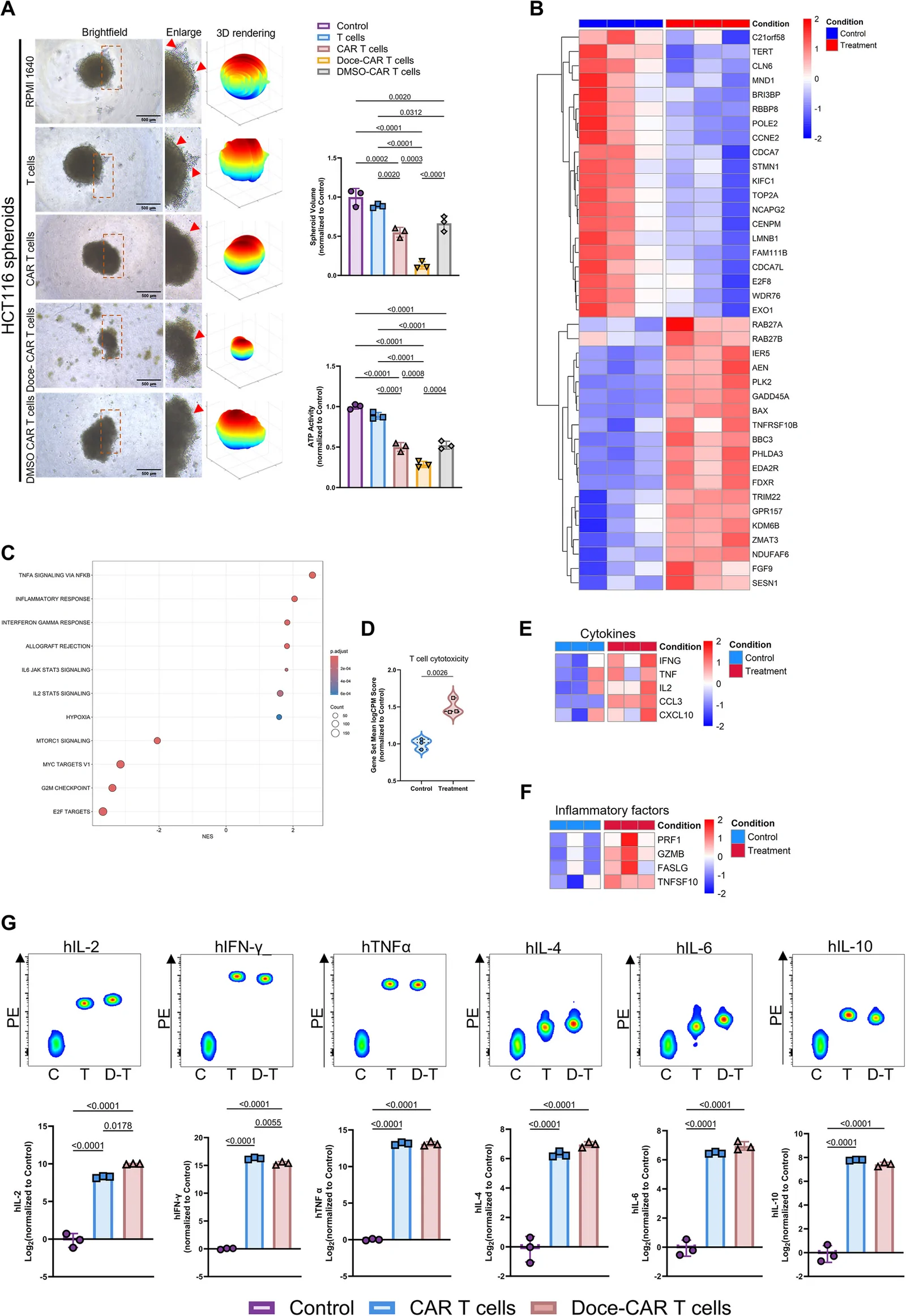
Evaluation of cytotoxicity, transcriptomic profiling, and cytokine secretory profiles in CAR T cells under docetaxel priming conditions. **A** Representative brightfield microscopy images, 3D volumetric reconstructions, and quantitative analysis of relative spheroid volume and metabolic ATP activity in HCT116 tumor spheroids following co-culture with media only. (RPMI 1640), unprimed T cells, conventional CAR T cells, Doce-CAR T cells, or vehicle control (DMSO-CAR T cells). Red arrows indicate areas of morphological alteration. **B** Heatmap showing differential gene expression profiles between control and docetaxel-primed CAR T cells, including cell cycle and vesicle trafficking genes. **C** Dot plot showing enriched signaling pathways derived from GSEA analysis of transcriptomic data. **D** Gene set expression scores for T cell cytotoxicity signatures, calculated as the normalized mean logCPM of constituent genes in control versus Doce-CAR T cells. **E**, **F** Heatmaps displaying the relative expression levels (LogCPM) of selected cytokine genes (**E**), as well as cytolytic effector genes and death receptor ligands (**F**). **G** Multiplex bead-based quantification of cytokine concentrations (hIL-2, hIFN-γ, hTNF-α, hIL-4, hIL-6, and hIL-10) in culture supernatants. (For **A**, **D**, and **G**, data are presented as the mean ± SEM of n = 3 biological replicates. Statistical significance was determined by one-way ANOVA followed by Tukey’s multiple comparison test for A and G, or unpaired Student’s t-test for D.)

Doce-CAR T cells induced regression of HCT116 tumor spheroids (Fig. 4A). This increased activity was also observed against mesothelin-negative tumor cell lines (MCF-7 and NCI-H460; Fig. S4A, B) as well as the mesothelin-expressing gastric carcinoma cell line HGC27 (Fig. S4C), while non-malignant HEK293T cells remained largely unaffected (Fig. S4D). The effect was not attributable to residual drug, as direct docetaxel exposure had minimal impact on established HCT116 spheroids under the same conditions (Fig. S4E–G). These findings suggest the presence of an antigen-independent cytotoxic component following docetaxel priming, potentially mediated by enhanced expression of death ligands such as FasL and TRAIL; however, the precise mechanisms underlying this non-antigen-specific activity remain to be fully characterized.

Transcriptomic analysis was consistent with concurrent changes in proliferative and effector pathways. Genes involved in cell division were broadly downregulated, whereas the vesicle trafficking regulator RAB27A and multiple cytotoxic effector genes, including PRF1, GZMB, FASLG, and TNFSF10, were upregulated (Fig. 4B–F). Expression patterns of secreted cytokines were also altered in treated cells (Fig. 4E, G). Protein-level analysis confirmed increased cytotoxic capacity. Doce-CAR T cells showed higher surface expression of FasL (Fig. S5A) and TRAIL (Fig. S5B), together with increased intracellular Granzyme B (Fig. S5C) and Perforin (Fig. S5D).

Despite this enhanced effector profile, proliferation-associated programs were reduced. Gene Set Variation Analysis (GSVA) demonstrated broad repression of proliferation signatures (Fig. S5E) and lower E2F activity scores (Fig. S5F). This reduction in cell cycle activity was not accompanied by increased apoptosis, as apoptosis scores remained stable (Fig. S5G) alongside upregulation of the pro-survival gene BCL2 (Fig. S5H). Consistent with earlier transcriptional data, these features were associated with inhibition of the G2M checkpoint (Fig. 3I) and induction of CDKN1A (p21) (Fig. 3J).

Beyond enhancing cytotoxic effector programs, docetaxel also profoundly rewired cellular metabolism. To functionally validate this transcriptomic profile and examine the potential for metabolic-cycle uncoupling across T cell subsets [21, 22], orthogonal validations were performed (Fig. S6). MET-flow analysis revealed increased expression of the primary glucose transporter 1 (GLUT1) across all analyzed CAR T cell sub-populations upon docetaxel treatment (Fig. S6A–D), whereas the protein expression of the rate-limiting enzyme hexokinase 1 (HK1) remained largely unchanged across these subsets (Fig. S6E–H). Functional metabolic assays further showed a significantly increased glucose consumption rate in Doce-CAR T cells compared to proliferating controls on a per-cell basis (Fig. S6I). Crucially, this increased glucose acquisition was uncoupled from proportional lactate production, which was significantly reduced (Fig. S6J). Together, these findings support a model in which docetaxel enhances glucose utilization without promoting a classical fermentative glycolytic phenotype, providing functional evidence for metabolic-cycle uncoupling.

### Primed CAR T cells mediate tumor control with associated stromal changes and systemic persistence

**Fig. 5:**
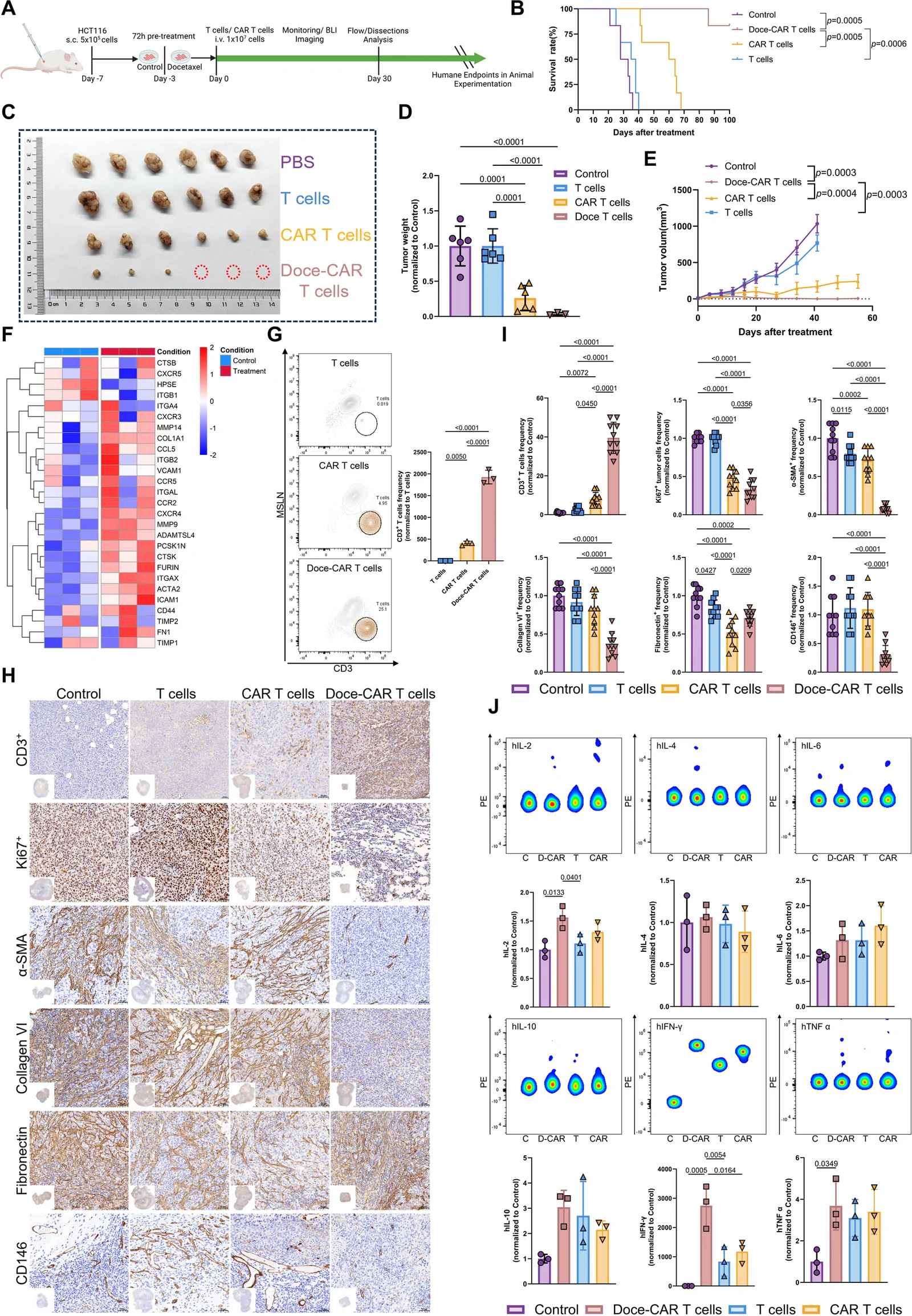
Evaluation of in vivo antitumor efficacy and tumor microenvironment remodeling following adoptive transfer of Doce-CAR T cells. **A** Schematic illustration of the experimental design and treatment timeline for the HCT116 subcutaneous xenograft model. Prior to adoptive transfer, CAR T cells were primed ex vivo with 10 nM docetaxel for 72 h and thoroughly washed before infusion. **B** Kaplan–Meier survival analysis of tumor-bearing mice treated with PBS (Control), unmodified T cells, conventional CAR T cells, or Doce-CAR T cells. **C**, **D** Assessment of terminal tumor burden, showing macroscopic images of excised tumors at the study endpoint (**C**) and quantitative analysis of tumor weight (normalized to Control) (**D**). **E** Longitudinal monitoring of tumor volume kinetics over the observation period. **F** Heatmap derived from RNA-seq data displaying the relative expression levels (LogCPM) of selected genes associated with chemotaxis, cellular migration, and extracellular matrix degradation. **G** Representative flow cytometry contour plots and quantification of intratumoral CD3⁺ T cell frequency within the tumor digests. **H**, **I** Immunohistochemical (IHC) evaluation of resected tumor tissues. Representative micrographs (**H**) and quantitative density analysis (**I**) of T cell infiltration (CD3), tumor proliferation (Ki67), stromal fibroblasts (α-SMA), extracellular matrix proteins (Collagen VI, Fibronectin), and endothelial cells (CD146). **J** Multiplex bead-based flow cytometric profiling and quantification of intratumoral cytokine concentrations (hIL-2, hIL-4, hIL-6, hIL-10, hIFN-γ, hTNF-α) from tissue homogenates.(Data are presented as the mean ± SEM of n ≥ 3 biological replicates/animals. Statistical significance was determined by the log-rank (Mantel-Cox) test for B, and one-way ANOVA followed by Tukey’s multiple comparison test for multi-group analyses in **D**, **E**, **G**, **I**, and **J**)

Under the treatment schema employing a split-cohort design (Fig. 5A), Doce-CAR T cells prolonged overall survival in the longitudinal observation cohort (Fig. 5B). To assess intermediate TME remodeling prior to severe tumor ulceration, a parallel dissection cohort was evaluated at Day 30 post-treatment. In this cohort, Doce-CAR T cells significantly reduced tumor burden, as reflected by lower terminal tumor weights (Fig. 5C, D). Longitudinal measurements of the survival cohort showed limited tumor regrowth in the treated group (Fig. 5E). BLI (Fig. S7A) demonstrated comparable initial tumor establishment between groups (Fig. S7B), followed by signal expansion in controls but suppression in mice receiving Doce-CAR T cells (Fig. S7C).

Transcriptional analysis of treated CAR T cells revealed increased expression of extracellular matrix metalloproteinases (MMPs), such as MMP9 and MMP14, and chemokine receptors associated with tissue trafficking (CXCR4, CCR5; Fig. 5F). Consistent with these changes, intratumoral CAR T cell accumulation was increased by more than fivefold within the viable cell population of the digested tumor tissues (Fig. 5G). Histological assessment of viable tumor regions showed reduced normalized staining for stromal and matrix components, including α-SMA, Collagen VI, and Fibronectin, in tumors from the Doce-CAR T cell group (Fig. 5H, I). Consistent with strategies to overcome physical exclusion [18, 23], these changes were accompanied by increased CD3⁺ lymphocyte density and higher intratumoral levels of IL-2, IFN-γ, and TNF-α in the tissue homogenates (Fig. 5J). Although MMP upregulation at the transcriptional level is consistent with enhanced stromal penetration capacity, a direct causal relationship between MMP expression and the observed increase in intratumoral accumulation was not formally established in this study.

Peripheral lymphoid organs showed evidence of expanded T cell compartments. Spleens from treated mice were enlarged (Fig. S7D) with increased spleen index (Fig. S7E) and lymphoid hyperplasia on histology (Fig. S7F). T cells isolated from these mice showed elevated expression of cytotoxic effectors, including surface FasL (Fig. S7G) and TRAIL (Fig. S7H), together with increased intracellular Perforin and Granzyme B and higher IFN-γ production (Fig. S7I–L).

Phenotypic analysis indicated enrichment of less differentiated subsets in vivo. Both CD4⁺ and CD8⁺ compartments showed increased proportions of SCM and CM cells (Fig. S8A–D). Expression of inhibitory and senescence-associated markers was reduced. PD-1 and CD57 levels were lower across multiple differentiation states, including EM and EMRA subsets (Fig. S9A–H). This potent efficacy was achieved with a favorable safety profile, as evidenced by intact organ histology (Fig. S10A), stable body weight (Fig. S10B), and normal liver function (Fig. S10C).

To further evaluate the generalizability of docetaxel priming across distinct tumor histologies, we established an independent xenograft model using the MSLN-expressing gastric carcinoma cell line HGC27 (Fig. S11A). In this model, Doce-CAR T cells significantly suppressed tumor growth (Fig. S11B), prolonged overall survival (Fig. S11C), and maintained stable body weight throughout treatment (Fig. S11D). These findings recapitulate the therapeutic benefit observed in the HCT116 model and support the robustness of this priming strategy across different solid tumor contexts.

### Docetaxel priming is associated with altered vesicle secretion contributing to paracrine antitumor activity

Recent work has reported taxane-induced cytotoxicity mediated by extracellular vesicles [17]. To test whether vesicular secretion contributes to the enhanced potency of primed cells, we inhibited exosome biogenesis using a neutral sphingomyelinase inhibitor (NSMi). Inhibition reduced cytotoxic effects in primed T cells (*p* = 0.0193) and CAR T cells (*p* = 0.0031), with increased tumor cell viability compared with vehicle controls (Fig. S12A, B). These results support a functional role for exosome secretion in antitumor activity.

Re-analysis of proteomic datasets showed upregulation of trafficking proteins and selective enrichment of vesicle-associated components, classified by Gene Ontology as exosome-related (Fig. S12C–G). In primed primary T cells, released vesicles displayed altered biophysical properties, including Zeta potential shift, with maintained size distributions (~ 100 nm; Fig. S12H–J). These vesicles showed penetration of tumor spheroids (Fig. S12K) and reduced spheroid integrity and metabolic activity (Fig. S12L–N), compared with vesicles from unprimed controls (Fig. S12O–Q).

**Fig. 6:**
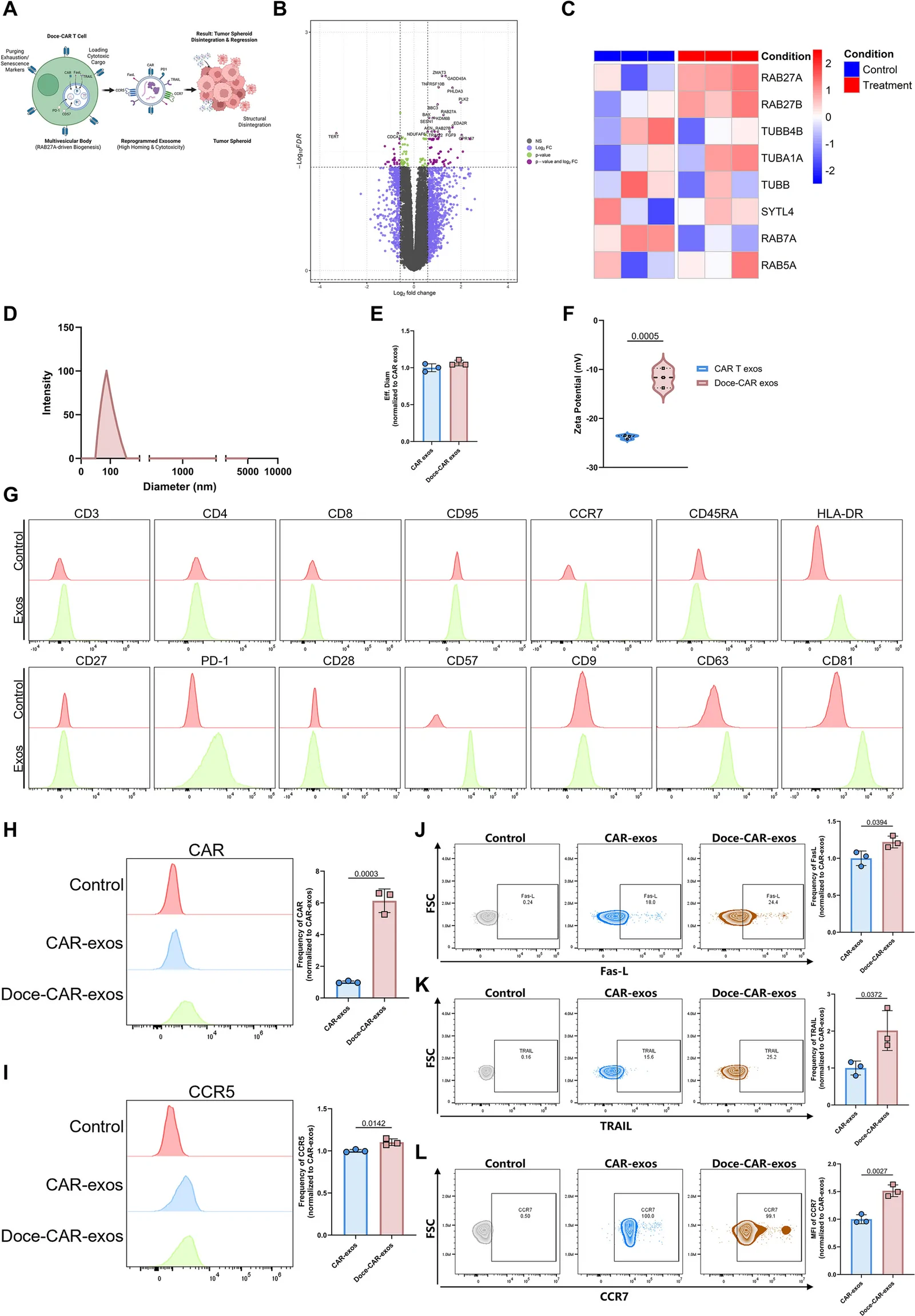
Transcriptomic profiling of exosome biogenesis pathways and biophysical/phenotypic characterization of secreted exosomes. **A** Schematic illustration of the proposed "Trash-to-Treasure" secretion model, depicting the hypothesized docetaxel-induced, RAB27A-driven biogenesis program and targeted cargo sorting. **B** Volcano plot derived from RNA-seq analysis of Doce-CAR T cells versus control cells, visualizing the global differential gene expression landscape. **C** Heatmap displaying the relative expression levels (Z-score) of selected genes involved in vesicle trafficking and exosome biogenesis (e.g., RAB27A, RAB27B, SYTL4). **D**–**F** Biophysical characterization of secreted vesicles, showing a representative particle size distribution profile (**D**), quantitative analysis of the mean effective diameter normalized to CAR exosomes (**E**), and comparative analysis of surface Zeta potential (mV) (**F**). **G** Flow cytometric profiling of the vesicular surface proteome. Representative histograms display the relative expression of lineage markers (CD3, CD4, CD8), functional and memory-associated receptors (CD95, CCR7, CD45RA, HLA-DR, CD27, CD28), exhaustion/senescence markers (PD-1, CD57), and canonical exosome markers (CD9, CD63, CD81) on vesicles derived from control CAR T cells versus Doce-CAR T cells. **H**–**L** Flow cytometric quantification of functional cargo markers on CAR exosomes versus Doce-CAR exosomes. Data are presented as representative histograms or contour plots (left) and normalized frequency or mean fluorescence intensity (MFI) quantification (right) for the CAR construct (**H**), the tissue-homing receptor CCR5 (**I**), the cytotoxic ligand FasL (**J**), the cytotoxic ligand TRAIL (**K**), and the lymphoid-homing receptor CCR7 (**L**). (For **E** and **H**–**L**, data are presented as the mean ± SEM of *n* = 3 biologically independent samples. For F, data are presented as a violin plot with individual data points. Statistical significance was determined by an unpaired Student’s t-test.)

Transcriptomic profiling of primed CAR T cells revealed a coordinated induction of vesicle trafficking genes associated with exosome biogenesis, including RAB27A and RAB27B (Fig. 6A, B), as well as SYTL4 (Fig. 6C). Biophysical characterization indicated released vesicles with preserved size (~ 100 nm; Fig. 6D, E) and altered surface charge (Fig. 6F). Proteomic analysis of isolated vesicles showed enrichment of PD-1 and CD57 (Fig. 6G), the CAR protein (*p* = 0.0003; Fig. 6H), CCR5 (Fig. 6I), FasL (Fig. 6J), TRAIL (Fig. 6K), and CCR7 (Fig. 6L).

**Fig. 7:**
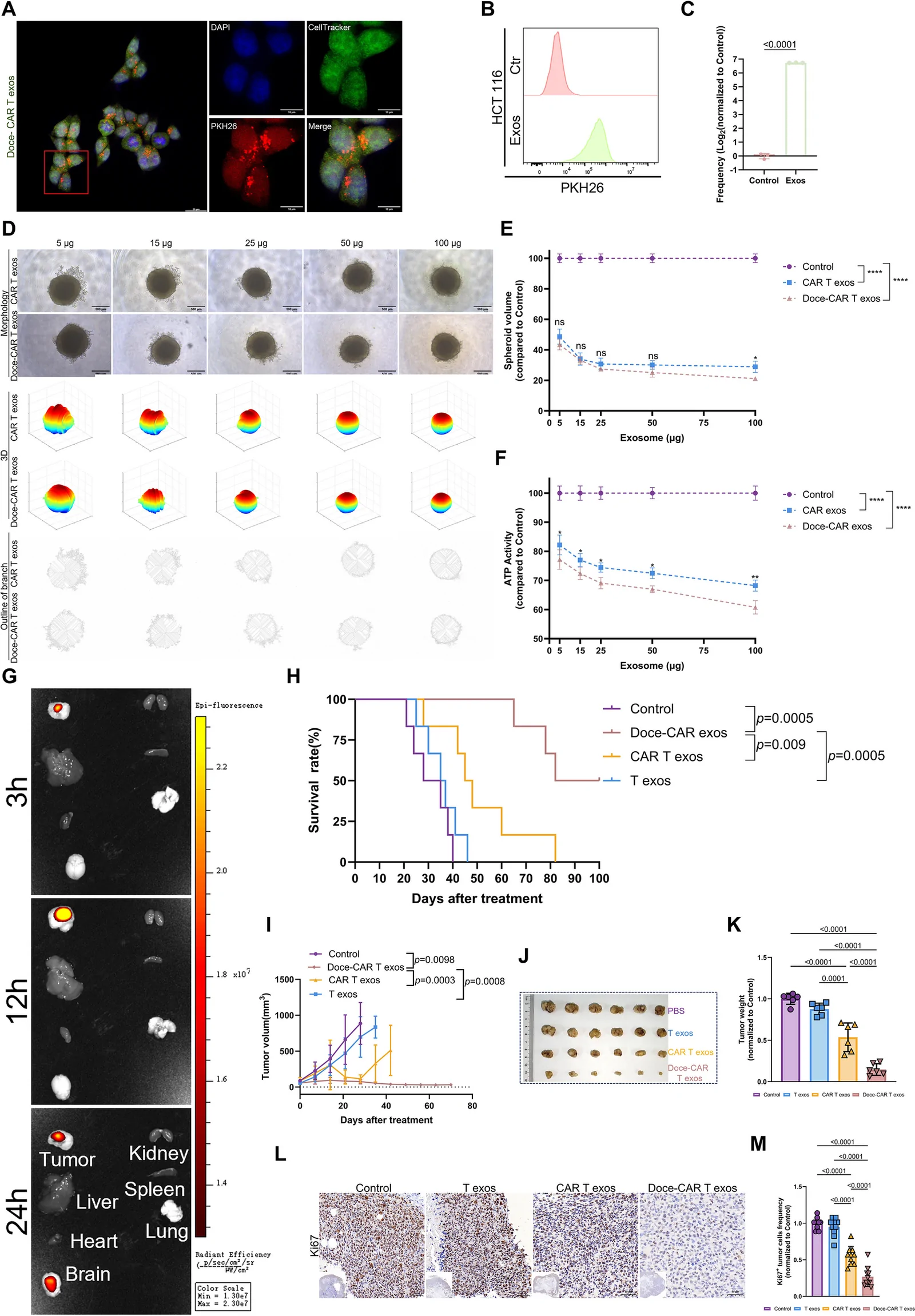
In vitro and in vivo evaluation of tumor targeting and therapeutic efficacy of Doce-CAR exosomes. **A** Representative confocal microscopy images of HCT116 tumor cells following incubation with PKH26-labeled Doce-CAR exosomes (red). Cells were counterstained with CellTracker Green and DAPI (blue). **B**, **C** Flow cytometric quantification of exosome internalization. Representative histograms of PKH26 fluorescence (**B**) and quantitative analysis of the frequency of PKH26⁺ cells (Log₂ normalized to Control) (**C**). **D** Representative brightfield morphology (top), 3D surface plot reconstructions (middle), and invasion branch outlines (bottom) of HCT116 spheroids treated with graded doses (5–100 µg) of CAR exosomes versus Doce-CAR exosomes. **E**, **F** Dose-dependent quantitative analysis of relative spheroid volume (**E**) and metabolic ATP activity (**F**) following exosome treatment. **G** Ex vivo biodistribution kinetics. Representative fluorescence images of major organs (Tumor, Kidney, Spleen, Liver, Heart, Lung, Brain) harvested from tumor-bearing mice at 3 h, 12 h, and 24 h post-injection. **H** Kaplan–Meier survival analysis of tumor-bearing mice treated with PBS (Control), T cell exosomes (T exos), CAR exosomes, or Doce-CAR exosomes. **I** Longitudinal tumor volume kinetics measured over the treatment course. **J**, **K** Assessment of endpoint tumor burden, showing macroscopic images of excised tumors (**J**) and quantitative analysis of tumor weight (normalized to Control) (**K**). **L**, **M** Immunohistochemical evaluation of tumor proliferation. Representative micrographs of tumor sections stained for Ki67 (**L**) and quantitative analysis of the frequency of Ki67⁺ tumor cells (normalized to Control) (**M**). (Data are presented as the mean ± SEM of *n* = 3 biologically independent samples for in vitro assays, and *n* = 6 animals per group for in vivo experiments. Statistical significance was determined by an unpaired Student’s t-test for C, the log-rank test for H, and one-way ANOVA followed by appropriate multiple comparison tests for K, M, as well as for the endpoint analysis of **E**, **F**, and **I**)

Doce-CAR T-derived exosomes (Doce-CAR exos) were internalized by HCT116 cells, as shown by confocal microscopy with perinuclear accumulation (Fig. 7A) and quantified by flow cytometry (Fig. 7B, C). Exposure reduced spheroid structure and metabolic activity (Fig. 7D–F). In vivo imaging of DiR-labeled vesicles indicated accumulation in tumor xenografts within 3 h (Fig. S13A, B), with retention relative to clearance organs (Fig. 7G).

Solid tumor lines (HCT116, MKN45, MCF-7, NCI-H460, HGC27) expressed death receptor 5 DR5 (TRAIL-R2) and Fas (Fig. S13C, D). CAFs also expressed these receptors. Administration of Doce-CAR exos reduced tumor growth (Fig. S13E–G) and prolonged survival (Fig. 7H), with lower residual tumor burden (Fig. 7I–K) and stable body weight (Fig. S13H). Treated tumors showed reduced Ki67 staining (Fig. 7L, M).

These data indicate that docetaxel priming is associated with altered secretory activity and selective cargo enrichment in exosomes, contributing to bystander antitumor effects in vitro and in vivo.

### Doce-CAR exos are associated with changes in recipient T cell composition and function

**Fig. 8:**
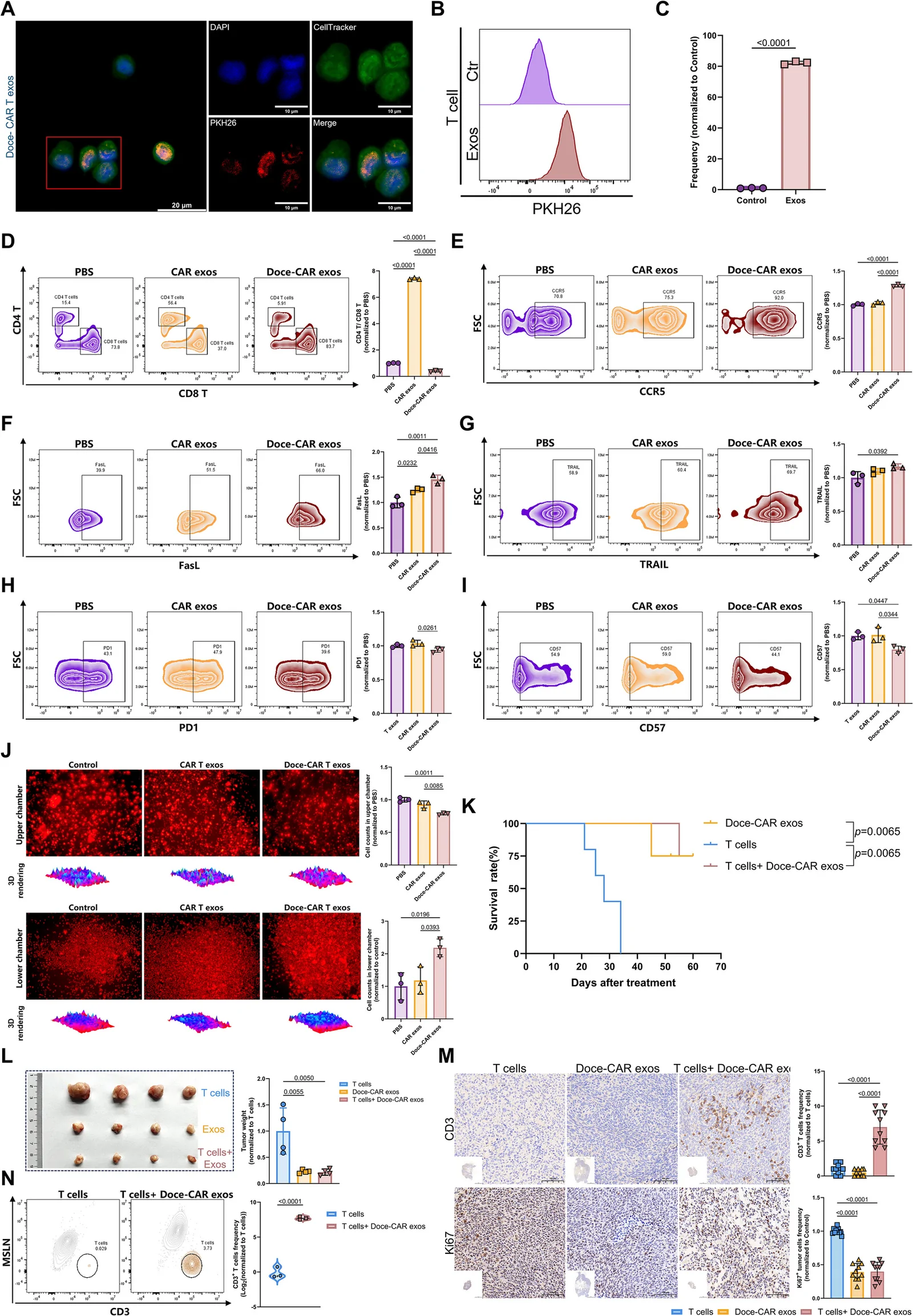
Phenotypic modulation of recipient T cells and in vivo evaluation of combinatorial therapy with Doce-CAR exosomes. **A** Representative confocal microscopy images of CellTracker-stained recipient T cells (green) following incubation with PKH26-labeled Doce-CAR exosomes (red). Nuclei were counterstained with DAPI (blue). **B**, **C** Flow cytometric evaluation of exosome internalization, showing representative histograms of PKH26 fluorescence (**B**) and quantitative analysis of the frequency of PKH26⁺ recipient T cells (normalized to Control) (**C**). **D** Flow cytometric assessment of T cell lineage distribution. Representative flow cytometry contour plots and quantitative analysis of the CD4⁺/CD8⁺ ratio (normalized to PBS) in recipient T cells following exosome co-culture. **E**–**I** Flow cytometric profiling of recipient T cells. Representative contour plots (left) and normalized quantitative analysis (right) of the expression of the tissue-homing receptor CCR5 (**E**), cytotoxic ligands FasL (**F**) and TRAIL (**G**), and coinhibitory/senescence receptors PD-1 (**H**) and CD57 (**I**). **J** Transwell migration assay evaluating T cell chemotaxis following treatment with PBS, CAR exosomes, or Doce-CAR exosomes. Representative fluorescence images of the upper and lower chambers, corresponding 3D surface renderings, and quantitative cell counts are shown. **K** Kaplan–Meier survival analysis of HCT116 tumor-bearing mice treated with unmodified T cells alone, Doce-CAR exosomes alone, or a combinatorial regimen (T cells + Doce-CAR exos). **L** Assessment of terminal tumor burden, displaying macroscopic images of excised tumors and quantitative analysis of tumor weight (normalized to the T cells group). **M** Immunohistochemical (IHC) evaluation of resected tumor tissues. Representative micrographs stained for T cell infiltration (CD3) and tumor proliferation (Ki67), with corresponding quantitative frequency analysis. **N** Flow cytometric quantification of intratumoral T cell infiltration. Representative contour plots and quantification of CD3⁺ cell frequency (Log₂ normalized to the T cells group) within the tumor digests.(For A–J, data are presented as the mean ± SEM of *n* = 3 biologically independent samples. For K–N, data are presented as the mean ± SEM of *n* = 4 animals per treatment group. Statistical significance was determined by an unpaired Student’s t-test for C, the log-rank test for K, and one-way ANOVA followed by Tukey’s multiple comparison test for D–J and L–N)

To examine whether Doce-CAR exos influence recipient T cells, we first assessed vesicle uptake. Confocal microscopy showed intracellular accumulation of PKH26-labeled exosomes within the cytoplasm of CellTracker-labeled T cells (Fig. 8A). Flow cytometry confirmed a uniform positive fluorescence shift compared with untreated controls (p < 0.0001; Fig. 8B, C), indicating efficient transfer of vesicular material into T cells.

Functional analysis revealed altered subset composition following exposure to primed exosomes. In contrast to conventional CAR exosomes (CAR exos), which favored CD4⁺ cells, primed exosomes increased the relative abundance of CD8⁺ T cells (Fig. 8D). This shift was accompanied by changes in migration-associated receptors. CCR5 expression was increased (Fig. 8E) and extended to more than 96% of CD8⁺ T cells (Fig. S14A, B), while expression of the lymphoid homing receptor CCR7 was preserved (Fig. S14C). Co-expression of CCR5 and CCR7 is consistent with the capacity of T cells to traffic between peripheral tissues and lymphoid organs, a feature described in circulating memory T cell populations [24]. Furthermore, division tracking showed divergent proliferative responses, with an increased division index in CD8⁺ cells and reduced proliferation of CD4⁺ cells (Fig. S14D).

High-dimensional phenotyping further indicated changes in differentiation state. UMAP analysis showed enrichment of populations in both CD4⁺ and CD8⁺ compartments, with reduced representation of EMRA cells compared with controls (Fig. S14E–H). The expanded CD27⁺CD28⁺CCR7⁺ subsets correspond to SCM cells that have been associated with long-term immune persistence and antitumor activity [25].

These phenotypic changes were accompanied by increased expression of cytotoxic effector ligands. FasL and TRAIL were upregulated following exosome exposure (Fig. 8F, G), whereas expression of exhaustion- and senescence-associated markers PD-1 and CD57 remained low (Fig. 8H, I). Reduced PD-1 and CD57 expression was most evident within CD8⁺ SCM and CM subsets (Fig. S15A–F).

In vitro Transwell assays showed increased T cell migration in response to primed exosomes (Fig. 8J). In vivo, co-administration of primed exosomes with T cells prolonged survival (Fig. 8K), decreased terminal tumor burden (Fig. 8L), and reduced overall tumor growth (Fig. S16A–D), which was associated with increased intratumoral T cell accumulation (Fig. 8M, N).

Systemically, treated mice developed splenomegaly (Fig. S16E, F). This immune expansion occurred without detectable toxicity, as body weight and liver indices remained within normal ranges (Fig. S16G, H). Phenotypically, there was an enrichment of SCM and CM subsets showing low exhaustion marker expression (Fig. S16I–L), and splenic T cells displayed upregulation of CCR5 together with FasL and TRAIL (Fig. S16M–O).

Checkpoint and senescence markers were reduced in splenic T cells following treatment. Heatmap and violin plot analyses demonstrated decreased PD-1 and CD57 expression (Fig. S17A-C), particularly within CD8⁺ SCM and CM subsets (Fig. S17D–G).

These systemic changes are consistent with prior studies showing that exosomal cargo can influence the balance between T cell exhaustion and memory formation [26]. Together with reports that exosome-associated signals can modulate immune activation [27, 28], the present findings indicate that primed CAR T-derived exosomes are associated with redistribution and functional changes in host T cell populations, contributing to antitumor effects.

## Discussion

CAR T cell therapies have achieved clinical success in hematologic malignancies; however, their efficacy in solid tumors remains limited by the immunosuppressive microenvironment, physical stromal barriers, and functional exhaustion [29]. Current strategies to address these limitations often involve complex genetic engineering to enhance persistence and function [30]. In parallel, pharmacological modulation during cell manufacturing has been explored as a scalable approach to preserve less differentiated T cell states [30–32].

Our retrospective analysis of breast cancer patients treated with docetaxel provided evidence for an immunological component to treatment benefit. Patients with low NLR showed prolonged survival compared with those with high NLR (116 vs. 93 months; *p* = 0.036), and absolute lymphocyte count was associated with outcomes (*p* = 0.043). These findings suggest an association between host lymphocyte reserve and docetaxel efficacy, consistent with reports that lymphocyte-rich tumors may derive greater benefit from taxanes [33].

This observation led us to evaluate the direct effects of low-dose docetaxel on T cell phenotypes. Exposure to sub-cytotoxic concentrations was associated with constrained proliferation and maintained effector function, with features consistent with stem-like memory traits. Transcriptomic analysis indicated downregulation of cell cycle pathways, including E2F targets and G2M checkpoint signatures, mediated by CDKN1A (p21) upregulation [34, 35]. Concurrently, expression of memory-associated transcription factors (TCF7, LEF1) was increased, while markers of terminal differentiation (PRDM1, TBX21) were reduced. Metabolic and survival genes, including MYC and BCL2, were upregulated. These changes were accompanied by decreased expression of inhibitory markers PD-1 and CD57. This phenotype is consistent with prior observations that taxanes can influence T cell secretory pathways [17, 36]. The findings indicate that low-dose docetaxel priming alters differentiation and transcriptional programs in CAR T cells, supporting enhanced antitumor responses in solid tumor.

We propose that low-dose docetaxel redirects cellular resources away from proliferation and toward effector synthesis and secretion. This functional shift aligns with the “Metabolic-Cycle Uncoupling” state defined in our results. Our functional metabolic validations reveal that pharmacological cell cycle blockade by low-dose docetaxel shifts CAR T cells into a distinct metabolic state, consistent with the paradigm recently outlined by Van Haften et al. [37], in which cell cycle-arrested CD8 + T cells exhibit enhanced nutrient acquisition and altered utilization patterns to support effector functions. While vehicle-treated control CAR T cells exhibit classical activation-induced glycolytic dynamics [38], Doce-CAR T cells maintain high glucose utilization independent of active clonal expansion. Based on our MET-Flow and functional flux analyses (Supplementary Figure S6), this increased glucose uptake occurs without a proportional increase in lactate production, suggesting a possible uncoupling from classical fermentative glycolysis. By uncoupling nutrient utilization from cell cycle progression, docetaxel pre-arms CAR T cells with favorable metabolic adaptations to endure the hostile solid tumor microenvironment. Prompted by reports of non-canonical vesicle release [17], transcriptomic profiling revealed upregulation of vesicle-trafficking regulators, including RAB27A and RAB27B [39], suggesting increased exosome biogenesis following priming. Thus, rather than supporting sustained cell division, the metabolic and transcriptional remodeling observed after docetaxel exposure appears mechanistically linked to enhanced secretory output.

Analysis of vesicle cargo and surface markers is consistent with a regulated sorting process. Surface exhaustion and senescence markers that decreased on primed T cells were detectable in isolated vesicle preparations, consistent with redistribution of these proteins into the secreted fraction. Building on prior reports that exosomal checkpoint molecules can modulate PD-1/PD-L1 interactions [12, 26], we consider it plausible that vesicle-associated PD-1 could have functional consequences in the TME; however, the precise impact of exosomal PD-1 on PD-L1 signaling in vivo requires direct functional testing.

Concurrently, exosomal cargo was enriched for effector molecules, including FasL, TRAIL, and notably the CAR protein itself [40, 41]. These observations indicate that Doce-CAR T cells release exosomes that simultaneously shed inhibitory markers (PD-1, CD57) from the parent cell while delivering cytotoxic ligands, CAR receptors, and homing molecules to recipient cells. This coordinated secretory pathway suggests a dual-modality therapeutic system executing a synergistic, two-pronged attack. First, exosomes armed with CAR, FasL, and TRAIL directly induce target cell apoptosis. Concurrently, exosomes deliver homing receptors (CCR5) and shed exhaustion/senescence markers (PD-1/CD57), systemically expanding host SCM/CM compartments [41]. Given the immense solid tumor burden, this exosome -mediated bystander licensing may represent an important contributor to antitumor efficacy. Furthermore, our findings delineate the complementary positioning of parental CAR T cells and their exosomes. While infused Doce-CAR T cells act as autonomous “living factories” that continuously proliferate and secrete exosomes in situ, the isolated exosomes serve as an independent, potent cell-free therapeutic platform. Although requiring repetitive dosing, this cell-free modality offers distinct translational advantages, including mitigated cytokine release syndrome risks, predictable pharmacokinetics, and highly scalable off-the-shelf manufacturing potential.

A key advantage of this strategy lies in the therapeutic potential of the secreted exosomes. Whereas exosomes from unmodified immune cells, such as γδ T cells [41], exhibit antitumor activity primarily through broad-spectrum ligands [42–44], docetaxel priming enhances Rab27-dependent exosome biogenesis and selectively enriches cargo with antigen-specific CAR and death receptor ligands. This dependence on the secretory pathway was mechanistically confirmed by significant attenuation of cytotoxicity upon inhibition of exosome biogenesis with a NSMi (*p* = 0.0031) [45].

Furthermore, this approach overcomes stromal barriers in solid tumors. Primed CAR T cells upregulated matrix-degrading enzymes (MMP9, MMP14), promoting intratumoral infiltration and remodeling of the desmoplastic niche. In parallel, the exosomes targeted CAFs, which highly express Fas and DR5 [46, 47]. Enrichment of FasL and TRAIL in exosomes [48] enabled the simultaneous elimination of tumor cells and stromal components, resulting in structural disruption of the immunosuppressive microenvironment in vitro and superior efficacy in vivo. Collectively, low-dose docetaxel priming establishes a scalable, non-genetic strategy that reprograms CAR T cells and their secreted exosomes into a coordinated system capable of overcoming multiple barriers in solid tumors, with direct integrability into existing manufacturing protocols.

Finally, this strategy addresses a fundamental limitation of adoptive cell therapy: the finite number of infused cells relative to the extensive solid tumor burden. Docetaxel-primed exosomes extend beyond direct cytotoxicity by modulating host T-cell function. Robust internalization of these exosomes by recipient T cells was confirmed (p < 0.0001), supporting active intercellular transfer [49, 50]. Primed exosomes induced expression of tissue-homing receptor CCR5 and preserved lymphoid-homing receptor CCR7 in bystander T cells [51, 52].

This paracrine modulation shifted systemic T-cell differentiation toward enrichment of SCM subsets in lymphoid organs, with concomitant downregulation of PD-1 and CD57. Given the critical role of SCM cells in long-term antitumor immunity [19], these changes promote sustained responses. This effect is mechanistically distinct from the immunosuppressive role of tumor-derived exosomal PD-L1 [27, 28], as primed exosomes—through PD-1 shedding and delivery of activating cargo—enhance host immune competence [53].

While our study establishes a framework for pharmacologically potentiating CAR T cells, limitations warrant rigorous consideration. First, reliance on immunodeficient xenograft models precludes a comprehensive assessment of potential off-target toxicities, particularly exosome-induced systemic inflammation in the context of a fully competent immune system [54]. Second, the generalizability of docetaxel priming across different CAR designs, target antigens, and tumor histologies requires further established validation beyond our current MSLN-targeting construct. Third, the clinical cohort analysis is retrospective, single-center, and limited to 98 patients; the observed association between NLR and survival outcomes cannot establish causality and cannot be directly extrapolated to the manufactured CAR T cell product context. Fourth, while our orthogonal MET-flow analyses provide robust single-cell functional validation for metabolic-cycle uncoupling, mapping the absolute global metabolic flux in vivo warrants future investigation. Fifth, the long-term in vivo persistence and susceptibility to secondary exhaustion remain incompletely characterized. Sixth, the technical caveat of ultracentrifugation-based isolation means effects should be attributed to the mixed small extracellular vesicle fraction rather than purely canonical exosomes. Finally, bridging this strategy to the clinic demands rigorous GMP-compatible manufacturing standardization—potentially leveraging automated closed-system technologies—to address scalability and donor-dependent variability [55].

## Conclusions

Despite these constraints, this pharmacological strategy offers distinct translational advantages over complex gene-editing approaches. By avoiding the genotoxic risks and regulatory complexities associated with additional genetic manipulation [1], docetaxel priming functions as a metabolic modulation step compatible with existing Good Manufacturing Practice (GMP) workflows. Ultimately, this study proposes a preclinical proof-of-concept for a manufacturing paradigm utilizing a transient pharmacological cue to generate a CAR T cell product with enhanced functional properties, including evidence of stromal remodeling and paracrine immune modulation in xenograft models. This approach suggests a potential shift toward a more multi-faceted therapeutic strategy, though clinical translation will require further mechanistic validation, manufacturing optimization, and evaluation in immunocompetent models.

## Supplementary Information

Supplementary Material 1. Supplemental Table 1. Baseline clinicopathological characteristics of breast cancer patients stratified by pretreatment absolute lymphocyte count (LY).

Supplementary Material 2. Supplemental Table 2. Baseline clinicopathological characteristics of breast cancer patients stratified by pretreatment Neutrophil-to-Lymphocyte Ratio (NLR).

Supplementary Material 3. Supplemental Table 3. Baseline clinicopathological characteristics of breast cancer patients stratified by pretreatment Lymphocyte-to-Monocyte Ratio (LMR).

Supplementary Material 4. Supplemental Figure 1. In vitro evaluation of paclitaxel-mediated effects on primary T cells and characterization of docetaxel-primed MSLN-CAR Jurkat T cells. (A) Quantitative viability analysis of primary T cells following exposure to a concentration gradient of paclitaxel (2–14 nM), assessed by CCK-8 assay. (B) Flow cytometric evaluation of T cell proliferation. Representative histograms (left) and quantitative analysis of the relative mean fluorescence intensity (MFI) (right) of Ki67 in unmodified T cells versus paclitaxel-primed T cells (Pacli-T). (C) Representative brightfield microscopy images, 3D volumetric surface reconstructions, and quantitative analyses of relative spheroid volume and metabolic ATP activity in HCT116 spheroids following co-culture with media control (RPMI 1640), unmodified T cells, docetaxel-primed T cells (Doce-T), or paclitaxel-primed T cells (Pacli-T). Red arrows indicate areas of morphological alteration. (D) Schematic representation of the second-generation anti-Mesothelin (MSLN) CAR construct comprising the Anetumab-derived scFv, CD8 hinge and transmembrane domains, 4-1BB costimulatory domain, and CD3ζ signaling domain. (E) Viability analysis of MSLN-CAR Jurkat T cells treated with a concentration gradient of docetaxel (700–950 pM), assessed by Trypan blue exclusion. (F) Flow cytometric profiling of surface MSLN expression. Representative histograms are shown across a panel of human solid tumor cell lines (NCI-H460, MCF-7, MKN45, HCT116, HGC27) and cancer-associated fibroblasts (CAFs). (G) Quantitative analysis of in vitro cytotoxicity of conventional CAR Jurkat T cells versus docetaxel-primed CAR Jurkat T cells (Doce-CAR Jurkat T) against HCT116 monolayers at Effector:Target (E:T) ratios of 3:1 and 10:1. (H) Representative brightfield microscopy images, 3D volumetric reconstructions, and quantitative analyses of relative spheroid volume and metabolic ATP activity in MSLN-positive HCT116 tumor spheroids following co-culture with media control (RPMI 1640), conventional CAR Jurkat T cells, or Doce-CAR Jurkat T cells. (I) Assessment of off-target cytotoxicity using MSLN-negative HEK293T spheroids. Representative images, 3D reconstructions, and quantification of relative spheroid volume and metabolic ATP activity following co-culture with media control versus Doce-CAR Jurkat T cells.(Data are presented as the mean ± SEM of n ≥ 3 biologically independent samples. Statistical significance was determined by one-way ANOVA followed by Tukey’s multiple comparison test for A, C, E, and H, or an unpaired Student’s t-test for B, G, and I).

Supplementary Material 5. Supplemental Figure 2. Viability, transcriptional signatures, and activation marker profiles of CAR T cells following low-dose docetaxel priming. (A) Representative flow cytometry plots assessing CAR transduction efficiency in primary human T cells. (B) Dose-titration analysis of primary MSLN-CAR T cells treated with a docetaxel concentration gradient (2–12 nM). Cell survival was assessed by Trypan blue exclusion (left) and overall metabolic fitness by viability assays (right). (C, D) Heatmaps derived from RNA-seq data displaying the relative expression of costimulatory and metabolic regulators (C: CD28, ICOS, MYC) and lymphoid-homing receptors (D: CCR7, SELL, PTPRC, IL7R, S1PR1) in CAR T cells versus Doce-CAR T cells. (E–I) Violin plots quantifying the specific gene expression levels (LogCPM) of CCR7 (E), SELL (F), PTPRC (G), IL7R (H), and S1PR1 (I). (J–M) Assessment of surface activation markers. Representative flow cytometric histograms and quantitative analysis of the relative expression frequency of CD25 (J, K) and CD69 (L, M) on conventional CAR T cells versus Doce-CAR T cells, evaluated at the identical time point corresponding to the RNA-seq profiling. (For B, E–I, K, and M, data are presented as the mean ± SEM of n = 3 biologically independent samples. Statistical significance was determined by one-way ANOVA followed by Tukey’s multiple comparison test for B, or an unpaired Student’s t-test for E–I, K, and M).

Supplementary Material 6. Supplemental Figure 3. Expression profiling of exhaustion and senescence markers in CAR T cells following low-dose docetaxel priming. (A) Heatmap derived from RNA-seq data displaying the relative expression levels of coinhibitory receptor genes (PDCD1, CTLA4, LAG3, TIGIT, HAVCR2) and the senescence-associated gene B3GAT1 in control versus docetaxel-primed CAR T cells. (B, C) Violin plots quantifying the frequency (Log₂ transformed) of surface PD-1⁺ (B) and CD57⁺ (C) cells within the total, CD4⁺, and CD8⁺ CAR T cell populations. (D, E) Heatmaps summarizing the mean expression frequency of PD-1⁺ (D) and CD57⁺ (E) cells across distinct CD4⁺ and CD8⁺ T cell differentiation subsets: Stem cell memory (SCM), Central memory (CM), Effector memory (EM), and Terminally differentiated effector memory (EMRA). (F–I) Detailed quantitative profiling showing the Log₂ transformed frequency of PD-1⁺ cells within specific CD4⁺ (F) and CD8⁺ (G) memory subsets, as well as CD57⁺ cells within CD4⁺ (H) and CD8⁺ (I) memory subsets.(For B, C, and F–I, n = 3 biologically independent samples per group. Data are presented as violin plots showing the distribution of individual biological replicates. Statistical significance was determined by an unpaired Student’s t-test).

Supplementary Material 7. Supplemental Figure 4. In vitro cytotoxicity evaluation of Doce-CAR T cells across diverse spheroid models and assessment of residual drug effects. (A, B) Representative brightfield microscopy images, 3D volumetric reconstructions, and quantitative analysis of relative spheroid volume and metabolic ATP activity in MSLN-negative MCF-7 breast carcinoma (A) and NCI-H460 lung carcinoma (B) spheroids following co-culture with media (RPMI 1640), conventional CAR T cells, or Doce-CAR T cells. Red arrows indicate areas of morphological alteration. (C) Representative brightfield microscopy images, 3D volumetric reconstructions, and quantitative analysis of relative spheroid volume and metabolic ATP activity in MSLN-expressing HGC27 gastric carcinoma spheroids following co-culture with media, unmodified T cells, conventional CAR T cells, or Doce-CAR T cells. (D) Safety assessment in non-malignant cell models. Representative images, 3D reconstructions, and quantification of spheroid volume and metabolic ATP activity in HEK 293T spheroids co-cultured with Doce-CAR T cells versus media control. (E, F) Pharmacological dose-response curves assessing cell survival to determine the IC50 of docetaxel in HCT116 cells cultured as 2D monolayers (E) versus 3D spheroids (F). (G) Functional assessment of direct pharmacological toxicity versus cell-mediated cytotoxicity. Representative images, 3D reconstructions, and quantitative analysis of HCT116 spheroids treated with media (Control), docetaxel-primed un-transduced T cells (Doce-T cells), Doce-CAR T cells, or 10 nM docetaxel alone.(For A–D and G, data are presented as the mean ± SEM of n = 3 biological replicates. Statistical significance was determined by one-way ANOVA followed by Tukey’s multiple comparison test for A, B, C, and G, or unpaired Student’s t-test for D).

Supplementary Material 8. Supplemental Figure 5. Profiling of cytotoxic effector expression and transcriptomic signatures of cell cycle and apoptosis following docetaxel priming. (A–D) Flow cytometric analysis of cytotoxic effector molecules. Representative contour plots and quantitative analysis of the relative expression of surface death ligands FasL (A) and TRAIL (B), as well as the mean fluorescence intensity (MFI) of intracellular cytolytic granules Granzyme B (C) and Perforin (D) in conventional CAR T cells versus Doce-CAR T cells. (E) Heatmap of Gene Set Variation Analysis (GSVA) scores comparing the global enrichment of the "Apoptosis", "G2M_Checkpoint", and "E2F_Targets" pathways between control and docetaxel-treated CAR T cells. (F, G) Violin plots quantifying the specific GSVA enrichment scores for the "E2F_Targets" (F) and "Apoptosis" (G) pathways. (H) Heatmap derived from RNA-seq data showing the relative expression levels (LogCPM) of selected apoptosis-associated genes (BAX, BCL2, CASP3, CASP9, BBC3).(For A–D, F, and G, n = 3 biologically independent samples per group. Data are presented as the mean ± SEM for bar graphs in A–D, or as violin plots showing the distribution of individual data points in F and G. Statistical significance was determined by an unpaired Student’s t-test).

Supplementary Material 9. Supplementary Figure S6. Flow cytometry-based protein expression profiling and extracellular metabolite turnover analysis of CAR T cells. (A–D) Flow cytometry analysis of glucose transporter 1 (GLUT1) expression across four distinct CAR T cell sub-populations following a 72-hour exposure to PBS (vehicle control) or 10 nM Docetaxel (Doce-CAR T). Representative histograms and summarized Mean Fluorescence Intensity (MFI) quantification are shown for each subset. (E–H) Flow cytometry analysis of Hexokinase 1 (HK1) expression across the corresponding CAR T cell sub-populations under identical treatment conditions. Summarized MFI quantification is presented for each indicated subset. (I) Extracellular glucose consumption rate quantified using a bioluminescent Glucose-Glo Assay over a 6-hour assay window. Rates were normalized to the assay duration and the integral of the viable cell count. (J) Extracellular lactate production rate measured via a bioluminescent Lactate-Glo Assay over the same 6-hour window under identical normalization parameters. n = 3 biological repeats. Data are presented as mean ± SEM. Statistical significance was determined by unpaired Student’s t-test (B-J).

Supplementary Material 10. Supplemental Figure 7. Longitudinal tumor monitoring and systemic immunological characterization of Docetaxel-primed CAR T cells. (A) Representative longitudinal bioluminescence imaging (BLI) of NSG mice bearing HCT116 tumors treated with PBS, T cells, CAR T cells, or Doce-CAR T cells. (B) Quantitative analysis of total bioluminescent flux at Day 0 (treatment initiation), confirming equivalent initial tumor engraftment across all experimental cohorts. (C) Longitudinal quantification of tumor burden dynamics (radiance, p/s/cm²/sr) over the 42-day observation period. (D, E) Macroscopic appearance of excised spleens (D) and quantification of the spleen index (E) at the study endpoint, serving as a proxy for systemic lymphoid expansion. (F) Representative Hematoxylin and Eosin (H&E) staining of resected tumor (top row) and spleen (bottom row) tissues showing histological architecture and cellular density. (G–L) Flow cytometric profiling of splenic CAR T cells quantifying the expression of surface death ligands (G, FasL; H, TRAIL), inflammatory cytokines (I, TNF-α; L, IFN-γ), and intracellular cytotoxic granules (J, Perforin; K, Granzyme B). (For A–F, n ≥ 5 biologically independent animals per group. For G–L, n = 3 biologically independent samples per group. Data are presented as the mean ± SEM. Statistical significance was determined by one-way ANOVA followed by Tukey’s multiple comparison test for B, E, and the endpoint analysis of C, and an unpaired Student’s t-test for G–L).

Supplementary Material 11. Supplemental Figure 8. High-dimensional single-cell phenotypic profiling of splenic T cell differentiation subsets following docetaxel priming. (A) Uniform Manifold Approximation and Projection (UMAP) visualization of splenic T cell populations clustered by differentiation phenotype (EM: Effector memory; CM: Central memory; EMRA: Terminally differentiated effector memory; SCM: Stem cell memory). (B) Feature plots projecting the expression intensity of canonical lineage (CD3, CD4, CD8) and differentiation markers (CCR7, CD45RA, CD27, PD-1, CD28, CD57) onto the UMAP space. (C, D) Representative UMAP plots highlighting specific cellular subsets (left) and quantitative violin plots (right) comparing the Log₂-transformed frequency of differentiation subsets within the CD4⁺ (C) and CD8⁺ (D) lineages across unmodified T cells, conventional CAR T cells, and Doce-CAR T cells. (For C and D, n = 3 biologically independent samples per group. Data are presented as violin plots showing the distribution of individual data points. Statistical significance was determined by one-way ANOVA followed by Tukey’s multiple comparison test).

Supplementary Material 12. Supplemental Figure 9. Systemic expression profiling of exhaustion and senescence markers across splenic T cell subsets following docetaxel priming. (A, B) Representative flow cytometry contour plots (left) and quantitative violin plots (right) detailing the Log₂-transformed frequency of the coinhibitory receptor PD-1⁺ (A) and the senescence marker CD57⁺ (B) in total splenic T cells across the indicated treatment groups. (C, D) Heatmap visualizations summarizing the mean expression frequency of PD-1⁺ (C) and CD57⁺ (D) cells across distinct CD4⁺ and CD8⁺ differentiation subsets (EM, CM, EMRA, SCM). (E–H) Detailed quantitative profiling of exhaustion and senescence markers stratified by lineage and memory status. Violin plots depict the Log₂-transformed frequency of PD-1⁺ cells within specific CD4⁺ (E) and CD8⁺ (F) subsets, alongside CD57⁺ cells within corresponding CD4⁺ (G) and CD8⁺ (H) subsets.(For A, B, and E–H, n = 3 biologically independent samples per group. Data are presented as violin plots showing the distribution of individual data points. Statistical significance was determined by one-way ANOVA followed by Tukey’s multiple comparison test).

Supplementary Material 13. Supplemental Figure 10. Evaluation of in vivo systemic safety and histological organ architecture following Doce-CAR T cell therapy. (A) Representative Hematoxylin and Eosin (H&E) stained sections of major vital organs (brain, heart, liver, lung, and kidney) harvested from tumor-bearing mice at the study endpoint. (B) Longitudinal monitoring of body weight kinetics for mice in the indicated treatment cohorts (Control, unmodified T cells, conventional CAR T cells, and Doce-CAR T cells) over the 56-day observation period. (C) Quantitative analysis of the liver index (defined as the ratio of liver weight to total body weight) at the study endpoint. Values are presented as normalized to the Control cohort.(Data are presented as the mean ± SEM of n = 5 biologically independent animals per treatment group. Statistical significance was determined by mixed-effects analysis for B, and one-way ANOVA followed by Tukey’s multiple comparison test for C).

Supplementary Material 14. Supplemental Figure 11. Evaluation of Doce-CAR T cell therapeutic efficacy and systemic tolerability in an independent HGC27 gastric carcinoma xenograft model. (A) Schematic illustration of the experimental design, tumor inoculation, and adoptive immunotherapy timeline in the subcutaneous HGC27 xenograft model. (B) Longitudinal monitoring of tumor volume kinetics (mm³) in tumor-bearing NSG mice following treatment with PBS (Control), unmodified T cells, conventional CAR T cells, or Doce-CAR T cells. (C) Kaplan-Meier survival estimates of HGC27 tumor-bearing mice across the indicated treatment cohorts. (D) Longitudinal quantification of individual mouse body weights (g) recorded over the post-treatment observation period. (For B and D, data are presented as the mean ± SEM of n = 5 animals per treatment group. Statistical significance was determined by the log-rank test for C, and one-way ANOVA followed by appropriate multiple comparison tests for the endpoint analysis of B and D).

Supplementary Material 15. Supplemental Figure 12. Pharmacological blockade of exosome secretion and proteomic/biophysical characterization of T cell-derived exosomes. (A, B) Quantitative analysis of metabolic ATP activity (normalized to Control) in HCT116 tumor cells co-cultured with docetaxel-primed unmodified T cells (A) or conventional CAR T cells (B) in the presence or absence of the neutral sphingomyelinase inhibitor (NSMi, GW4869) or DMSO vehicle control. (C–G) In silico re-analysis of external proteomic datasets. Volcano plots display global differential protein expression in total conditioned media (C) and purified extracellular vesicles (EVs) (D). (E) Venn diagram illustrating the overlap between the upregulated docetaxel-induced secretome and the Vesiclepedia database. (F) Quantification of upregulated protein cargo classified as EVs. (G) Gene Ontology (GO) enrichment analysis ranking the cellular components enriched in the secretome. (H–J) Biophysical characterization of exosomes isolated from unmodified T cells (T exos) and docetaxel-primed T cells (Doce-T exos). Representative particle size distribution profile (H), quantitative analysis of the mean effective diameter normalized to T exos (I), and a violin plot of surface Zeta potential (mV) measurements (J). (K) Representative confocal microscopy images demonstrating the internalization of PKH26-labeled Doce-T exosomes (red) by CellTracker-stained HCT116 cells (green). Nuclei were counterstained with DAPI (blue). (L) Representative brightfield microscopy, 3D volumetric surface reconstructions, and invasion branch outlines of HCT116 spheroids treated with graded doses (5–100 µg) of T exos versus Doce-T exos. (M, N) Quantitative analysis of metabolic ATP activity in spheroids treated with escalating doses of T exos (M) or Doce-T exos (N). (O–Q) Dose-response curves comparing the in vitro efficacy of Control (media), T exos, and Doce-T exos regarding relative metabolic ATP activity (O), spheroid volume (P), and invasive process length (Q). (For all quantitative panels, n = 3 biologically independent samples per group. Data are presented as the mean ± SEM for bar graphs and dose-response curves (A, B, I, O–Q), or as violin plots showing the distribution of individual data points (J, M, N). Statistical significance was determined by an unpaired Student’s t-test for I, J, and one-way ANOVA followed by Tukey’s multiple comparison test for A, B, M, N, and the endpoint dose analysis of O–Q).

Supplementary Material 16. Supplemental Figure 13. In vivo biodistribution profile of Doce-CAR exosomes, in vitro target receptor validation, and evaluation of systemic therapeutic efficacy. (A) Representative longitudinal whole-body fluorescence imaging of HCT116 tumor-bearing NSG mice at the indicated time points (3 h, 12 h, and 24 h) following intravenous injection of PBS (Control), DiR-labeled Doce-CAR exosomes (Exos-DiR), or free DiR dye (Free-DiR). (B) Representative in vivo fluorescence imaging demonstrating specific intratumoral accumulation. The merged panel (right) displays the spatial overlay of the bioluminescence signal from HCT116-Luciferase tumor cells (red) and the fluorescence signal from the targeted DiR-labeled exosomes (green). (C, D) Flow cytometric profiling of surface death receptors. Representative histograms display the baseline expression levels of DR5 (C) and Fas (D) across a comprehensive panel of human solid tumor cell lines (HCT116, MKN45, MCF-7, NCI-H460, HGC27) and cancer-associated fibroblasts (CAFs). (E) Quantitative analysis of total bioluminescent flux at Day 0 (treatment initiation) to assess baseline tumor engraftment equivalence across experimental cohorts. (F) Representative longitudinal whole-body bioluminescence imaging (BLI) of HCT116 tumor-bearing NSG mice treated with PBS (Control), T cell exosomes (T exos), CAR exosomes (CAR T exos), or Doce-CAR exosomes (Doce-CAR T exos) over a 42-day observation period. (G) Longitudinal quantification of tumor burden dynamics (radiance, p/s/cm²/sr) corresponding to the imaging cohorts in (F). (H) Longitudinal monitoring of body weight kinetics for the indicated treatment cohorts over the 42-day observation period. (For A, B, and E–H, n = 5 biologically independent animals per group. For C and D, data are representative of n = 3 independent in vitro experiments. Data are presented as the mean ± SEM. Statistical significance was determined by one-way ANOVA followed by appropriate multiple comparison tests for E, and for the endpoint analysis of G).

Supplementary Material 17. Supplemental Figure 14. Phenotypic, proliferative, and high-dimensional single-cell profiling of recipient T cells following exosome co-culture. (A, B) Flow cytometric analysis of the tissue-homing receptor CCR5 expression on recipient CD4⁺ (A) and CD8⁺ (B) T cells following co-culture with PBS, CAR exosomes, or Doce-CAR exosomes. Data are presented as representative contour plots (left) and normalized frequency quantification (right). (C) Quantitative analysis of the lymphoid-homing receptor CCR7 surface expression (Mean Fluorescence Intensity, MFI) on recipient T cells. (D) Assessment of lineage-specific proliferation kinetics. Representative proliferation dye dilution histograms (left) and division index quantification (right) for total T cells, CD4⁺ T cells, and CD8⁺ T cells. (E) Uniform Manifold Approximation and Projection (UMAP) visualization of recipient T cell populations clustered by differentiation phenotype (EM: Effector memory; CM: Central memory; EMRA: Terminally differentiated effector memory; SCM: Stem cell memory). (F) Feature plots projecting the single-cell expression intensity of canonical lineage (CD3, CD4, CD8) and differentiation markers (CD95, CCR7, CD45RA, CD27, PD-1, CD28, CD57) onto the UMAP space. (G, H) Representative UMAP plots highlighting specific cellular subsets (left) and quantitative violin plots (right) comparing the Log₂-transformed frequency of distinct differentiation subsets within the CD4⁺ (G) and CD8⁺ (H) lineages across the indicated treatment groups.(For all quantitative panels, n = 3 biologically independent samples per group. Data are presented as the mean ± SEM for bar graphs in A–D, or as violin plots showing the distribution of individual data points in G and H. Statistical significance was determined by one-way ANOVA followed by Tukey’s multiple comparison test).

Supplementary Material 18. Supplemental Figure 15. Expression profiling of exhaustion and senescence markers across recipient T cell differentiation subsets following exosome co-culture. (A, B) Heatmaps summarizing the mean expression frequency of the coinhibitory receptor PD-1 (A) and the senescence marker CD57 (B) across total and specific differentiation subsets (CM, EM, EMRA, SCM) of recipient CD4⁺ and CD8⁺ T cells following co-culture with PBS, CAR exosomes, or Doce-CAR exosomes. (C–F) Detailed quantitative evaluation of exhaustion and senescence markers. Violin plots depict the Log₂-transformed frequency of PD-1⁺ cells within total and specific CD4⁺ (C) and CD8⁺ (D) memory subsets, alongside the Log₂-transformed frequency of CD57⁺ cells within the corresponding CD4⁺ (E) and CD8⁺ (F) subsets, across the indicated treatment groups. (For C–F, n = 3 biologically independent samples per group. Data are presented as violin plots showing the distribution of individual data points. Statistical significance was determined by one-way ANOVA followed by Tukey’s multiple comparison test).

Supplementary Material 19. Supplemental Figure 16. Evaluation of in vivo combinatorial therapeutic efficacy, systemic safety, and splenic immune remodeling. (A–D) Longitudinal assessment of tumor burden in HCT116 tumor-bearing mice treated with unmodified T cells, Doce-CAR exosomes alone, or the combinatorial regimen (T cells + Doce-CAR exos). (A) Validation of baseline tumor engraftment equivalence at Day 0, showing quantitative analysis of normalized bioluminescence intensity. (B) Longitudinal kinetics of total bioluminescent flux (radiance, p/s/cm²/sr) over time. (C) Representative whole-body bioluminescence imaging (BLI) at the indicated time points (Days 0, 7, 14, 21, and 38). (D) Longitudinal monitoring of tumor volume kinetics (mm³). (E, F) Assessment of systemic lymphoid expansion. (E) Macroscopic appearance of excised spleens harvested at the study endpoint. (F) Quantitative analysis of the spleen index (normalized to Control) comparing the T cell and combinatorial treatment groups.(G, H) Systemic safety and toxicological evaluation. (G) Longitudinal body weight monitoring across the indicated treatment cohorts. (H) Quantitative analysis of the liver index (normalized to Control) at the study endpoint. (I, J) High-dimensional spectral profiling of splenic T cells. (I) Uniform Manifold Approximation and Projection (UMAP) visualization of splenic T cell populations clustered by differentiation phenotype. (J) Feature plots projecting the single-cell expression intensity of canonical lineage and differentiation markers onto the UMAP space. (K, L) Quantitative analysis of specific differentiation subsets within the splenic CD4⁺ (K) and CD8⁺ (L) compartments. Violin plots display the Log₂-transformed frequency of Effector memory (EM), Central memory (CM), Terminally differentiated effector memory (EMRA), and Stem cell memory (SCM) subsets between T cells and the combinatorial group. (M–O) Flow cytometric phenotypic characterization of the splenic T cell reservoir. Representative contour plots and quantification of the relative expression of FasL (M), TRAIL (N), and CCR5 (O) on splenic T cells. (For in vivo experiments, n = 4 biologically independent animals per group. Data are presented as the mean ± SEM for bar and line graphs (A, B, D, F, G, H, M–O), or as violin plots showing individual data points (K, L). Statistical significance was determined by one-way ANOVA followed by Tukey’s multiple comparison test for A, as well as for the endpoint analysis of B, D, and G; and an unpaired Student’s t-test for F, H, and K–O).

Supplementary Material 20. Supplemental Figure 17. Systemic expression profiling of exhaustion and senescence markers in the splenic compartment following combinatorial treatment. (A) Global assessment of splenic T cell exhaustion and senescence. Representative flow cytometry contour plots (left) and quantitative violin plots (right) comparing the Log₂-transformed frequency of PD-1⁺ (top) and CD57⁺ (bottom) splenic T cells. Within the quantitative plots, "Control" and "Exos" refer to the unmodified T cell monotherapy and the combinatorial regimen (T cells + Doce-CAR exos), respectively. (B, C) Heatmap visualizations summarizing the mean expression frequency of the coinhibitory receptor PD-1 (B) and the senescence marker CD57 (C) across total and distinct T cell differentiation subsets (CD4⁺ and CD8⁺; CM, EM, EMRA, SCM). (D–G) Detailed subset-specific quantitative analysis of exhaustion and senescence markers. Violin plots display the Log₂-transformed frequency of PD-1⁺ cells within total and specific CD4⁺ (D) and CD8⁺ (E) differentiation subsets, alongside the Log₂-transformed frequency of CD57⁺ cells within corresponding CD4⁺ (F) and CD8⁺ (G) subsets. (For all quantitative panels, n = 3 biologically independent samples per group. Data are presented as violin plots showing the distribution of individual data points. Statistical significance was determined by an unpaired Student’s t-test).

## Data Availability

All data generated in this study are available upon request to the lead contact.

## Abbreviations

BLI: Bioluminescence imaging
CAFs: Cancer-associated fibroblasts
CAR: Chimeric antigen receptor
CBC: Complete blood counts
CIs: Confidence intervals
CM: Central memory
CMC: Chemistry, Manufacturing, and Controls
Doce-CAR T cells: Docetaxel-primed CAR T cells
Doce-T: Docetaxel-treated T cells
DR5: Death receptor 5
E:T: Effector-to-target ratio
EM: Effector memory
EMRA: Terminally differentiated effector memory
FBS: Fetal bovine serum
FDR: False discovery rate
GMP: Good Manufacturing Practice
GO: Gene Ontology
GSEA: Gene Set Enrichment Analysis
GSVA: Gene Set Variation Analysis
H&E: Hematoxylin and Eosin
HK1: Hexokinase 1
HRs: Hazard ratios
IACUC: Institutional Animal Care and Use Committee
IHC: Immunohistochemistry
IRB: Institutional Review Board
LMR: Lymphocyte-to-monocyte ratio
MMP: Matrix metalloproteinase
MOI: Multiplicity of infection
MSLN: Mesothelin
NLR: Neutrophil-to-lymphocyte ratio
NSG: NOD scid gamma
NSMi: Neutral sphingomyelinase inhibitor
OS: Overall survival
PBMCs: Peripheral blood mononuclear cells
RLU: Relative luminescence units
scFv: Single-chain variable fragment
SCM: Stem cell memory
SPF: Specific pathogen-free
TME: Tumor microenvironment
ULA: Ultra-low attachment
