# Supplementary material for: Low-dose docetaxel reprograms CAR T cells for enhanced solid tumor immunity
Source: J Exp Clin Cancer Res. 2026 Jul 15;45:198. doi: 10.1186/s13046-026-03781-9 (PMC13584523; doi:10.1186/s13046-026-03781-9)
Supplement: Supplementary file 1 [file 13046_2026_3781_MOESM1_ESM.docx]

|  | **LY Groups** | | |  |
| --- | --- | --- | --- | --- |
| **Characteristic** | **Overall** N = 98*^1^* | **LY High (>1.11)** N = 77*^1^* | **LY Low (≤1.11)** N = 21*^1^* | **p-value***^2^* |
| **Age (Years)** | 50 (10) | 49 (10) | 53 (10) | 0.2 |
| **Overall Survival (Months)** | 104 (42) | 107 (42) | 91 (43) | 0.10 |
| **Survival Status** |  |  |  | 0.10 |
| Censored | 66 (67%) | 55 (71%) | 11 (52%) |  |
| Event (Death) | 32 (33%) | 22 (29%) | 10 (48%) |  |
| **T Stage (T0/T1/T2 vs T3/T4)** |  |  |  | 0.3 |
| T0/T1/T2 | 86 (88%) | 69 (90%) | 17 (81%) |  |
| T3/T4 | 12 (12%) | 8 (10%) | 4 (19%) |  |
| **N Stage (N0 vs N+)** |  |  |  | 0.7 |
| N0 | 62 (63%) | 48 (62%) | 14 (67%) |  |
| N+ (N1-N3) | 36 (37%) | 29 (38%) | 7 (33%) |  |
| **M Stage (M0 vs M1)** |  |  |  | 0.4 |
| M0 (Non-metastatic) | 89 (91%) | 71 (92%) | 18 (86%) |  |
| M1 (Metastatic) | 9 (9.2%) | 6 (7.8%) | 3 (14%) |  |
| **Overall Stage (I/II vs III/IV)** |  |  |  | 0.2 |
| Stage I/II (Early) | 68 (69%) | 56 (73%) | 12 (57%) |  |
| Stage III/IV (Advanced) | 30 (31%) | 21 (27%) | 9 (43%) |  |
| **Ki-67 Status** |  |  |  | 0.7 |
| Ki-67 Low (<30%) | 41 (42%) | 33 (43%) | 8 (38%) |  |
| Ki-67 High (≥30%) | 57 (58%) | 44 (57%) | 13 (62%) |  |
| **NLR Status** |  |  |  | <0.001 |
| NLR Low (≤2.34) | 48 (49%) | 45 (58%) | 3 (14%) |  |
| NLR High (>2.34) | 50 (51%) | 32 (42%) | 18 (86%) |  |
| **LMR Status** |  |  |  | 0.085 |
| LMR High (>3.58) | 49 (50%) | 42 (55%) | 7 (33%) |  |
| LMR Low (≤3.58) | 49 (50%) | 35 (45%) | 14 (67%) |  |
| *^1^*Mean (SD); n (%) | | | | |
| *^2^*Wilcoxon rank sum test; Pearson's Chi-squared test; Fisher's exact test | | | | |

**Supplemental Table 1. Baseline clinicopathological characteristics of breast cancer patients stratified by pretreatment absolute lymphocyte count (LY).**
