# Supplementary material for: Low-dose docetaxel reprograms CAR T cells for enhanced solid tumor immunity
Source: J Exp Clin Cancer Res. 2026 Jul 15;45:198. doi: 10.1186/s13046-026-03781-9 (PMC13584523; doi:10.1186/s13046-026-03781-9)
Supplement: Supplementary file 2 [file 13046_2026_3781_MOESM2_ESM.docx]

**Supplemental Table 2. Baseline clinicopathological characteristics of breast cancer patients stratified by pretreatment Neutrophil-to-Lymphocyte Ratio (NLR)**

|  | **NLR Groups** | | |  |
| --- | --- | --- | --- | --- |
| **Characteristic** | **Overall** N = 98^1^ | **NLR Low (≤2.34)** N = 48^1^ | **NLR High (>2.34)** N = 50^1^ | **p-value^2^** |
| **Age (Years)** | 50 (10) | 50 (9) | 50 (11) | 0.7 |
| **Overall Survival (Months)** | 104 (42) | 116 (35) | 93 (46) | 0.036 |
| **Survival Status** |  |  |  | 0.004 |
| Censored | 66 (67%) | 39 (81%) | 27 (54%) |  |
| Event (Death) | 32 (33%) | 9 (19%) | 23 (46%) |  |
| **T Stage (T0/T1/T2 vs T3/T4)** |  |  |  | 0.2 |
| T0/T1/T2 | 86 (88%) | 44 (92%) | 42 (84%) |  |
| T3/T4 | 12 (12%) | 4 (8.3%) | 8 (16%) |  |
| **N Stage (N0 vs N+)** |  |  |  | 0.13 |
| N0 | 62 (63%) | 34 (71%) | 28 (56%) |  |
| N+ (N1-N3) | 36 (37%) | 14 (29%) | 22 (44%) |  |
| **M Stage (M0 vs M1)** |  |  |  | 0.5 |
| M0 (Non-metastatic) | 89 (91%) | 45 (94%) | 44 (88%) |  |
| M1 (Metastatic) | 9 (9.2%) | 3 (6.3%) | 6 (12%) |  |
| **Overall Stage (I/II vs III/IV)** |  |  |  | 0.11 |
| Stage I/II (Early) | 68 (69%) | 37 (77%) | 31 (62%) |  |
| Stage III/IV (Advanced) | 30 (31%) | 11 (23%) | 19 (38%) |  |
| **Ki-67 Status** |  |  |  | 0.7 |
| Ki-67 Low (<30%) | 41 (42%) | 21 (44%) | 20 (40%) |  |
| Ki-67 High (≥30%) | 57 (58%) | 27 (56%) | 30 (60%) |  |
| **LMR Status** |  |  |  | 0.4 |
| LMR High (>3.58) | 49 (50%) | 26 (54%) | 23 (46%) |  |
| LMR Low (≤3.58) | 49 (50%) | 22 (46%) | 27 (54%) |  |
| **LY Status** |  |  |  | <0.001 |
| LY High (>1.11) | 77 (79%) | 45 (94%) | 32 (64%) |  |
| LY Low (≤1.11) | 21 (21%) | 3 (6.3%) | 18 (36%) |  |
| 1Mean (SD); n (%) | | | | |
| 2Wilcoxon rank sum test; Pearson's Chi-squared test; Fisher's exact test | | | | |
