# Supplementary material for: Low-dose docetaxel reprograms CAR T cells for enhanced solid tumor immunity
Source: J Exp Clin Cancer Res. 2026 Jul 15;45:198. doi: 10.1186/s13046-026-03781-9 (PMC13584523; doi:10.1186/s13046-026-03781-9)
Supplement: Supplementary file 3 [file 13046_2026_3781_MOESM3_ESM.docx]

**Supplemental Table 3. Baseline clinicopathological characteristics of breast cancer patients stratified by pretreatment Lymphocyte-to-Monocyte Ratio (LMR).**

|  |  | **LMR Groups** | |  |
| --- | --- | --- | --- | --- |
| **Characteristic** | **Overall** N = 98*^1^* | **LMR High (>3.58)** N = 49*^1^* | **LMR Low (≤3.58)** N = 49*^1^* | **p-value***^2^* |
| **Age (Years)** | 50 (10) | 48 (10) | 51 (10) | 0.12 |
| **Overall Survival (Months)** | 104 (42) | 107 (42) | 100 (43) | 0.7 |
| **Survival Status** |  |  |  | 0.7 |
| Censored | 66 (67%) | 34 (69%) | 32 (65%) |  |
| Event (Death) | 32 (33%) | 15 (31%) | 17 (35%) |  |
| **T Stage (T0/T1/T2 vs T3/T4)** |  |  |  | 0.2 |
| T0/T1/T2 | 86 (88%) | 41 (84%) | 45 (92%) |  |
| T3/T4 | 12 (12%) | 8 (16%) | 4 (8.2%) |  |
| **N Stage (N0 vs N+)** |  |  |  | 0.036 |
| N0 | 62 (63%) | 26 (53%) | 36 (73%) |  |
| N+ (N1-N3) | 36 (37%) | 23 (47%) | 13 (27%) |  |
| **M Stage (M0 vs M1)** |  |  |  | >0.9 |
| M0 (Non-metastatic) | 89 (91%) | 45 (92%) | 44 (90%) |  |
| M1 (Metastatic) | 9 (9.2%) | 4 (8.2%) | 5 (10%) |  |
| **Overall Stage (I/II vs III/IV)** |  |  |  | 0.080 |
| Stage I/II (Early) | 68 (69%) | 30 (61%) | 38 (78%) |  |
| Stage III/IV (Advanced) | 30 (31%) | 19 (39%) | 11 (22%) |  |
| **Ki-67 Status** |  |  |  | 0.5 |
| Ki-67 Low (<30%) | 41 (42%) | 19 (39%) | 22 (45%) |  |
| Ki-67 High (≥30%) | 57 (58%) | 30 (61%) | 27 (55%) |  |
| **NLR Status** |  |  |  | 0.4 |
| NLR Low (≤2.34) | 48 (49%) | 26 (53%) | 22 (45%) |  |
| NLR High (>2.34) | 50 (51%) | 23 (47%) | 27 (55%) |  |
| **LY Status** |  |  |  | 0.085 |
| LY High (>1.11) | 77 (79%) | 42 (86%) | 35 (71%) |  |
| LY Low (≤1.11) | 21 (21%) | 7 (14%) | 14 (29%) |  |
| *^1^*Mean (SD); n (%) | | | | |
| *^2^*Wilcoxon rank sum test; Pearson's Chi-squared test; Fisher's exact test | | | | |
